# Triggering NETosis via protease-activated receptor (PAR)-2 signaling as a mechanism of hijacking neutrophils function for pathogen benefits

**DOI:** 10.1371/journal.ppat.1007773

**Published:** 2019-05-20

**Authors:** Danuta Bryzek, Izabela Ciaston, Ewelina Dobosz, Anna Gasiorek, Anna Makarska, Michal Sarna, Sigrun Eick, Magdalena Puklo, Maciej Lech, Barbara Potempa, Jan Potempa, Joanna Koziel

**Affiliations:** 1 Department of Microbiology, Faculty of Biochemistry, Biophysics and Biotechnology, Jagiellonian University, Krakow, Poland; 2 Department of Biophysics, Faculty of Biochemistry, Biophysics and Biotechnology, Jagiellonian University, Krakow, Poland; 3 Department of Periodontology, School of Dental Medicine, University of Bern, Bern, Switzerland; 4 Klinikum der Ludwig-Maximilians-Universität München, Medizinische Klinik und Poliklinik IV, Department of Nephrology, Munich, Germany; 5 Department of Oral Immunity and Infectious Diseases, University of Louisville School of Dentistry, University of Louisville, Louisville, Kentucky, United States of America; University of Massachusetts Medical School, UNITED STATES

## Abstract

Neutrophil-derived networks of DNA-composed extracellular fibers covered with antimicrobial molecules, referred to as neutrophil extracellular traps (NETs), are recognized as a physiological microbicidal mechanism of innate immunity. The formation of NETs is also classified as a model of a cell death called NETosis. Despite intensive research on the NETs formation in response to pathogens, the role of specific bacteria-derived virulence factors in this process, although postulated, is still poorly understood. The aim of our study was to determine the role of gingipains, cysteine proteases responsible for the virulence of *P*. *gingivalis*, on the NETosis process induced by this major periodontopathogen. We showed that NETosis triggered by *P*. *gingivalis* is gingipain dependent since in the stark contrast to the wild-type strain (W83) the gingipain-null mutant strain only slightly induced the NETs formation. Furthermore, the direct effect of proteases on NETosis was documented using purified gingipains. Notably, the induction of NETosis was dependent on the catalytic activity of gingipains, since proteolytically inactive forms of enzymes showed reduced ability to trigger the NETs formation. Mechanistically, gingipain-induced NETosis was dependent on proteolytic activation of protease-activated receptor-2 (PAR-2). Intriguingly, both *P*. *gingivalis* and purified Arg-specific gingipains (Rgp) induced NETs that not only lacked bactericidal activity but instead stimulated the growth of bacteria species otherwise susceptible to killing in NETs. This protection was executed by proteolysis of bactericidal components of NETs. Taken together, gingipains play a dual role in NETosis: they are the potent direct inducers of NETs formation but in the same time, their activity prevents *P*. *gingivalis* entrapment and subsequent killing. This may explain a paradox that despite the massive accumulation of neutrophils and NETs formation in periodontal pockets periodontal pathogens and associated pathobionts thrive in this environment.

## Author summary

Periodontitis, or gum disease, is characterized by chronic inflammation and erosion of the tooth-supporting tissues. The condition is fuelled by bacterial accumulation on the tooth surface below the gum line that resists the host innate immune response, including massive accumulation of neutrophils. Despite possessing a formidable array of bactericidal machineries, including neutrophil extracellular traps (NETs) formation whereby neutrophils release DNA-composed fibers decorated with bactericidal proteins and peptides to efficiently trap and kill bacteria. Nevertheless, neutrophils in periodontitis are unable to clear the infection due to the presence of key periodontal pathogens, including *Porphyromonas gingivalis*. This bacterium secretes a variety of virulence factors, including proteases (gingipains) that allow the organism to manipulate the host immune response to benefit the entire dysbiotic microbial community. Here, we describe a unique strategy whereby *P*. *gingivalis* trigger NET formation through gingipain-dependent cleavage of Protease Activated Receptor (PAR)-2 on the neutrophil surface. Importantly, NETs formed in this way are deficient in antibacterial activity but instead, supports bacterial growth due to degradation of bactericidal components by gingipains. This finding may explain a paradox that dysbiotic bacteria flourished in periodontal pockets in spite of massive accumulation of neutrophils and abundant NETs formation.

## Introduction

Neutrophils are primary effectors of the innate immune system against microbial pathogens. In addition to phagocytic killing, neutrophils also catch and kill microbes via an alternative mechanism known as neutrophil extracellular trap (NET) formation. NETs are networks composed of chromatin and neutrophil granule proteins with high bactericidal potential. They are thought to neutralize pathogens and create a barrier that prevents the spread of bacteria [1]. The formation of NETs in response to several species of microorganisms has been shown; however, the mechanisms of NET induction by pathogens are largely unknown [2]. Except for lipopolysaccharide (LPS) [1] and *Pseudomonas aeruginosa* flagellin [3], pathogen-associated molecular patterns (PAMPs) have not been reported to directly trigger NETosis. Even in the case of LPS- and flagellin-induced NET formation, the signaling mechanism remains unknown, since NETosis was independent of specific receptors for these PAMPs [Toll-like receptor (TLR)4 and TLR5, respectively] [3–5]. Therefore, it has been postulated that, as with other processes of immune system activation, NET generation can be induced by specific virulence factors. To date, this has been shown only for an M1 surface protein of Group A *Streptococcus* (GAS) [6] and the secreted *S*. *aureus* toxins; Panton-Valentine leukocidin (PVL) and leukotoxin GH (LukGH) [2, 7]. Apart from that, little is known about how bacteria trigger NET formation.

Periodontitis is a very common form of oral disease in which NETosis seems to play an important role [8]. The disease results from dysbiotic microbiota colonizing the tooth surface below the gums, which initiates and drives chronic inflammation in the periodontium, slowly but irreversibly eroding the tissues supporting the teeth [9]. Among the hundreds of bacterial species identified in the subgingival biofilm, *Porphyromonas gingivalis* is recognized as a major periodontal pathogen [10]. It secretes gingipains, cysteine proteases with Arg-X (RgpA and RgpB) and Lys-X (Kgp) specificity [11] that contribute to 85% of the proteolytic activity of *P*. *gingivalis* [12] and are present in gingival crevicular fluid (GCF) from infected periodontitis sites at concentrations exceeding 100 nM [13]. Gingipains are associated with the bacterial cell surface or secreted into the extracellular environment, either in outer membrane vesicles (OMVs) or as soluble mediators [14]. The latter forms are able to diffuse into tissues away from the subgingival bacterial biofilm [15].

Gingipains are primary, essential virulence factors of *P*. *gingivalis* that affect the life-span of immune cells, neutralize antimicrobial peptides and antibodies, and modulate the biological activity of cytokines and complement factors [16, 17]. This creates an inflammatory environment rich in nutrients, in which *P*. *gingivalis* and accompanying inflammophilic periodontitis-associated microbiota can thrive, resistant to the bactericidal activity of phagocytes, including neutrophils [18, 19].

Neutrophils are essential for homeostasis in periodontal tissues, and their deficit or functional insufficiency is responsible for progressing forms of periodontitis [20]. Conversely, uncontrolled activity of neutrophil proteases, along with excessive release of reactive oxygen species (ROS), can lead to destruction of the periodontal soft tissue and organic components of the alveolar bone [21]. Thus, neutrophil numbers and activity, including NET formation and clearance, need to be balanced. This balance seems to be severely disturbed in periodontitis, since NETs have been found in copious amounts in GCF [22], purulent crevicular exudates, and biopsies of the pocket epithelium of periodontitis patients [23, 24]. This clinically observed abundant NETosis is likely due to neutrophils interacting with the bacteria in periodontal pockets [25], but the precise mechanisms underlying NET formation in periodontitis still need to be elucidated.

In this work, we investigated the interaction between *P*. *gingivalis* and neutrophils and found that gingipains can directly induce NET generation *in vitro* by hijacking the protease-activated receptor-2 (PAR-2) signaling pathway. Importantly, however, gingipain-induced NETs were deficient of bactericidal activity and instead promoted bacterial growth. This finding could explain the paradox that, despite the high levels of neutrophil accumulation and NET formation, periodontal pathogens and associated pathobionts thrive in inflamed periodontal sites.

## Results

### *P*. *gingivalis* is a potent inducer of NET formation

**Fig 1:**
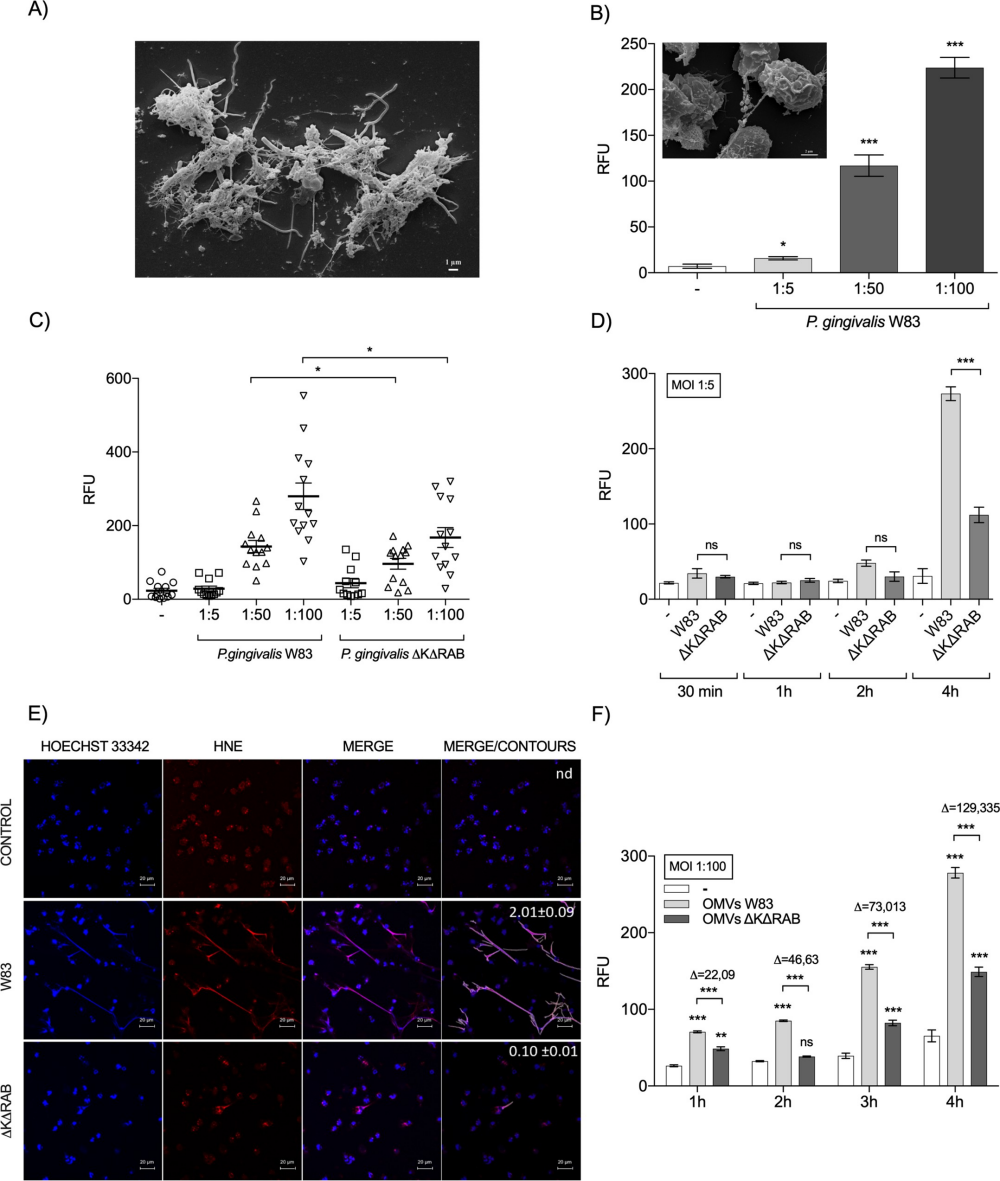
The generation of NETs by *P*. *gingivalis* is gingipain-dependent. (A) NETs visualized by SEM in GCF from patients with chronic periodontitis. (B) The generation of NETs by *P*. *gingivalis* W83 (MOI 1:5, 1:50, 1:100). The level of extracellular DNA released by neutrophils 1 h post-bacterial exposure was estimated by QPG. SEM visualization of *P*. *gingivalis* entrapped in NET structures induced by pathogens (W83) in neutrophils from healthy donors (insert). (C, D) Neutrophils were infected with *P*. *gingivalis* strains diametrically differing in the expression of gingipains (WT W83 and the gingipain-null ΔKΔRAB mutant) at MOIs of 1:5, 1:50, and 1:100 for 1 h (C), or at a MOI of 1:5 from 30 min to 4 h (D). The level of extracellular DNA was estimated by QPG. (E) Visualization of NETs by confocal laser scanning microscopy. DNA is shown in blue (Hoechst 33342) and human neutrophil elastase (HNE) is shown in red. Bars represent 20 μm. Quantitative analysis of NETs images was performed by merging blue and red channels (merge/contours). Percentage of the NET area in relation to the area of an image is presented as the mean value (± SEM) from three independent images; n.d.–NETs not detected. (F) OMVs isolated from W83 and the ΔKΔRAB mutant strains were incubated with neutrophils from 1 h to 4 h. The level of NETs was determined by QPG. Statistical significance was evaluated by unpaired t-test (B), two-way (C) and one-way (D, F) ANOVA, followed by Bonferroni’s multiple comparisons posttest. Mean data (± SEM) from 13 (C) or 3 (B, D, F) independent experiments using neutrophils from different healthy donors are shown. *P < 0.05, **P < 0.01, and ***P < 0.001; ns, non-significant.

Growing numbers of studies have detected NETs *in vivo*, especially in patients suffering from chronic inflammatory diseases [26–29]. The finding that NETs are abundant in the GCF suggests that they may contribute to the pathogenesis of periodontitis [22]. In the present study, we verified the presence of NETs in GCF samples collected from microbiologically examined, *P*. *gingivalis*-positive periodontitis patients. Scanning electron microscopy (SEM) analysis showed characteristic NET-like structures decorated with bacteria (Fig 1A). To determine the contribution of *P*. *gingivalis* to NET generation, neutrophils isolated from healthy donors were exposed to the viable pathogen. The level of released DNA was already significantly increased 1 h after infection of neutrophils, with the intensity of NET generation strongly dependent on the MOI (Fig 1B). NETosis was confirmed by SEM visualization of NET structures with entrapped *P*. *gingivalis* W83 (Fig 1B—insert). The phenomenon was not limited to the W83 strain, since neutrophil infection with other clinical and laboratory strains of *P*. *gingivalis* also effectively induced NET formation in a dose-dependent manner (S1 Fig). Collectively, these results confirm previous findings [25] that *P*. *gingivalis* strongly induces NET generation.

### The induction of NETs by *P*. *gingivalis* depends on gingipains

Despite the intensive study of NETosis in response to pathogens, the role of specific bacteria-derived virulence factors in this process remains poorly explored. After demonstrating that *P*. *gingivalis* is a potent inducer of NET release, we wished to elucidate the underlying mechanism. We focused on gingipains, cysteine proteases considered to be key virulence factors of *P*. *gingivalis* [17]. Initially, we compared the effects of the WT strain of *P*. *gingivalis* (W83), which expresses all three gingipains (RgpA, RgpB, and Kgp), with those of its isogenic mutant devoid of gingipain activity (ΔKΔRAB). In contrast to the WT strain (W83), the gingipain-null strain (ΔKΔRAB) was a weaker inducer of NET formation in both aerobic and anaerobic conditions. After 1 h, the difference was significant at a MOI of 1:50 and 1:100 but not at a MOI of 1:5 (Figs 1C and S2A). At a MOI of 1:5, the differences between the W83 and ΔKΔRAB strains with respect to their ability to induce NET formation were significant only at 4 h post-infection (Fig 1D). The formation of NETs was visualized using confocal microscopy to examine the co-localization of DNA with neutrophil elastase (NE) and the level of NETs was quantified (Fig 1E).

The association between NET formation and gingipain expression was confirmed using another gingipain-null mutant in the ATCC 33277 background (KDP 136) (S2B Fig) and OMVs. Of note, OMVs are predominant carriers of gingipains into gingival tissue at *P*. *gingivalis*-infected periodontitis sites [30]. As shown in Fig 1F, OMVs isolated from W83 showed a greater ability to form NETs than OMVs isolated from the gingipain-null isogenic mutant. Taken together, these data demonstrate that the induction of NETs by *P*. *gingivalis* is triggered by active gingipains in a manner largely independent of other bacterial cell surface appendages such as fimbriae.

### Purified gingipains are direct inducers of NETosis

**Fig 2:**
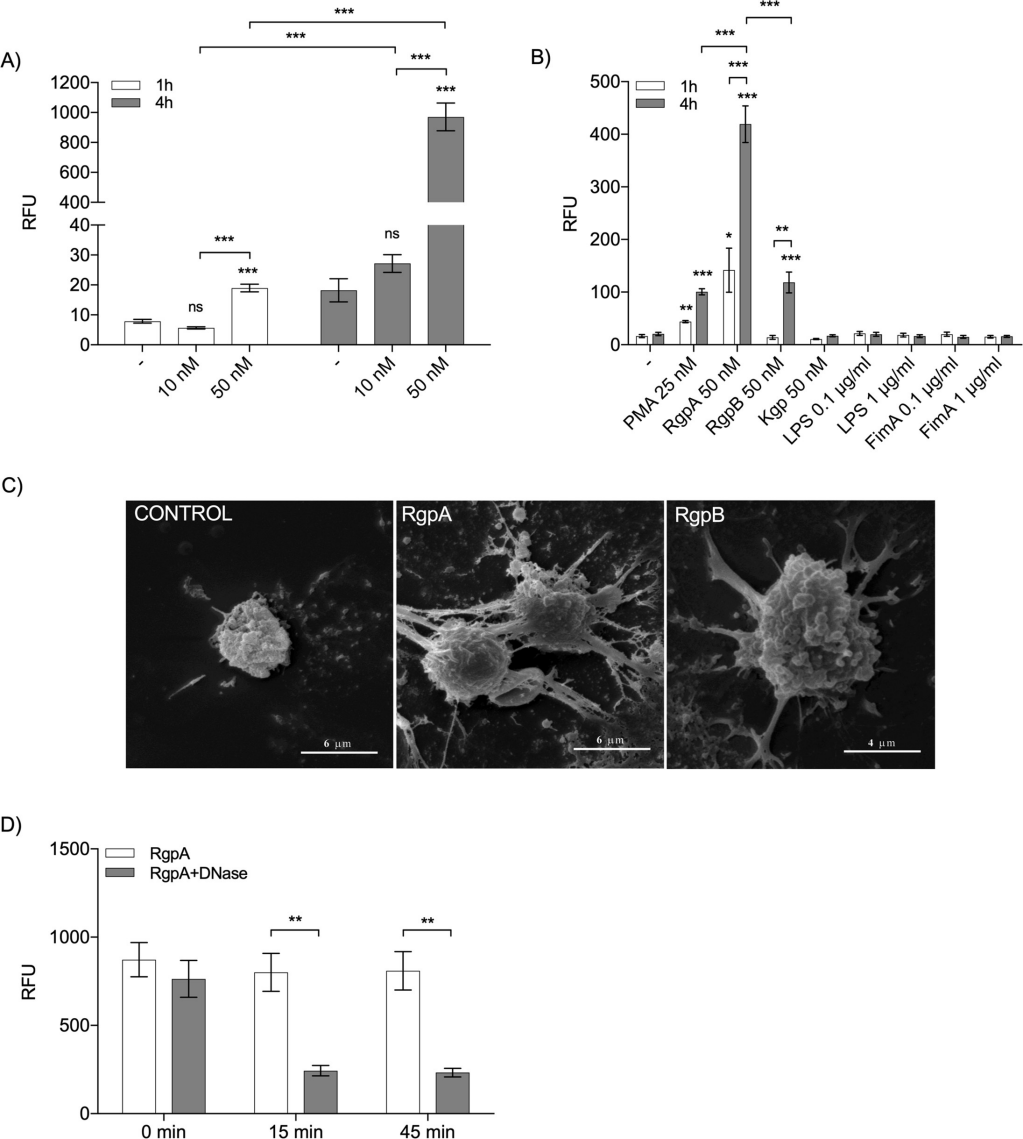
Purified gingipains promote NET generation. (A) The level of NETs induced by gingipain cocktails containing each enzyme at 10 or 50 nM after 1 or 4 h of incubation, as determined by QPG. (B) Isolated neutrophils were stimulated with different gingipains (RgpA, RgpB, or Kgp; 50 nM), LPS and FimA (each at 0.1 or 1 μg/ml), or 25 nM PMA, as a control for NET generation. The level of NETs was determined by QPG. (C) NET structures visualized by SEM after 4 h of incubation with 10 nM Arg-X gingipains (RgpA and RgpB). (D) Degradation of the DNA backbone of the NETs induced for 4 h with 50 nM RgpA. Collected NETs were incubated with DNase I (50 μg/ml) for 0, 15, or 45 min. (A, B, D) Statistical significance was evaluated by two-way ANOVA, followed by Bonferroni’s multiple comparisons posttest. Data are the mean (± SEM) from three separate experiments. *P < 0.05, **P < 0.01, and ***P < 0.001; ns, non-significant.

After determining the contribution of gingipains to *P*. *gingivalis*-induced NET formation, we wished to verify the direct role of these enzymes in the observed NETosis. In initial experiments, a mixture of both arginine-specific (RgpA, RgpB) and lysine-specific (Kgp) gingipains was used at a final concentration 10 or 50 nM per gingipain. SEM showed the typical DNA fibers extruding from fresh neutrophils treated with gingipains (S3A Fig). Next, the NET structure was visualized using confocal microscopy to examine the co-localization of DNA with neutrophil elastase (NE) with subsequent quantification [1, 31] (S3B Fig). The gingipain cocktail induced NET formation in a dose- and time-dependent manner (Fig 2A).

To quantify the ability of individual gingipains to trigger NETosis, neutrophils were treated with specific purified gingipains. Fluorimetric analysis of released fibers of DNA showed that, in contrast to Kgp, both arginine-specific proteases (RgpA and RgpB) induced NETs (Fig 2B). Out of these two gingipains, RgpA was a far more potent NET inducer. RgpA caused a significant release of DNA after only 1 h, and the amount of extruded DNA fibers tripled after an additional 3 h of incubation. By comparison, the equivalent dose of RgpB failed to trigger NETosis at early time points, while at 4 h, the level of extracellular DNA was approximately 25% of that induced by RgpA at the same time point (Fig 2B). Remarkably, the NET-inducing activity of RgpA was four times higher than that of PMA.

The presence of NET structures upon gingipain stimulation was confirmed by SEM imaging (Fig 2C). The DNA released upon gingipain treatment of neutrophils was susceptible to degradation by DNase I (Fig 2D). The unique role of gingipains in triggering NETosis was confirmed by examination of other *P*. *gingivalis* virulence factors, including LPS and major fimbriae (FimA), none of which exerted any significant effect on NET formation regardless of the incubation time (Fig 2B).

**Fig 3:**
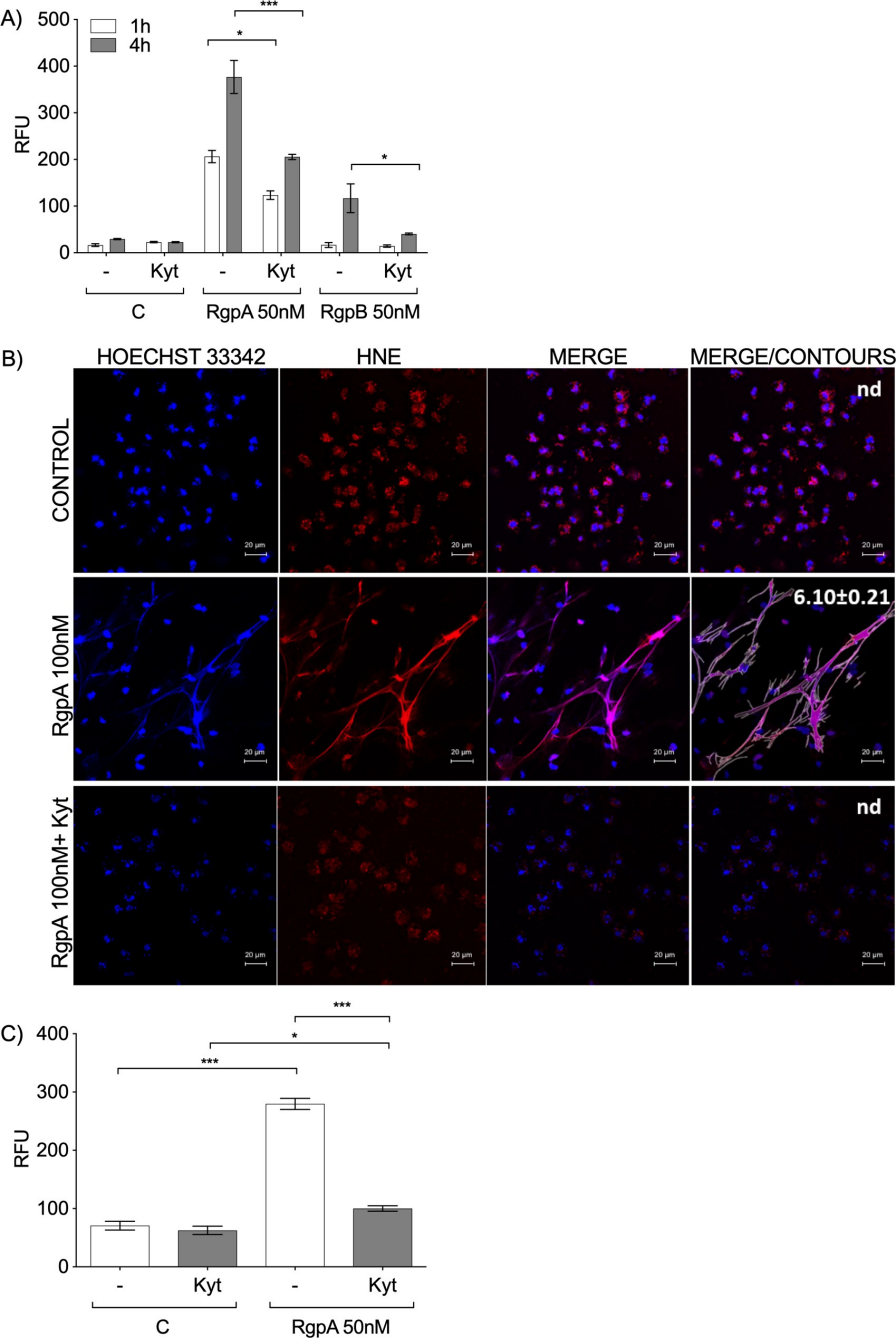
The role of the proteolytic activity of gingipains in NET formation. Human peripheral blood neutrophils (A) and neutrophils isolated from mouse bone marrow (C) were stimulated with 50 nM RgpA and/or RgpB in the presence or absence of Kyt-1 at a final concentration of 1 μM. The level of NETs was estimated by QPG at 1 h (A) and 4 h (A, C) after enzyme exposure. (B) Confocal laser scanning microscopy of NETs generated by human neutrophils, DNA is shown in blue (Hoechst 33342) and human neutrophil elastase (HNE) expression is shown in red. Bars represent 20 μm. Quantitative analysis of NETs images was performed by merging blue and red channels (merge/contours). Percentage of the NET area in relation to the area of an image is presented as the mean value (± SEM) from three independent images; n.d.–NETs not detected. (A, C) Statistical significance was evaluated by one-way ANOVA, followed by Bonferroni’s multiple comparisons posttest. Mean data (± SEM) from three independent experiments are shown. *P < 0.05 and ***P < 0.001.

Since *P*. *gingivalis* triggers NETosis in a gingipain-dependent manner, it is expected that gingipains must be proteolytically active to exert this effect. To verify this assumption, Kyt-1, a highly specific, reversible, non-toxic inhibitor of gingipains R [32], was used. Preincubation of gingipains with Kyt-1 significantly reduced, but did not completely eliminate, the ability of RgpA and RgpB to release DNA from human neutrophils (Figs 3A and 3B). Interestingly, the effect of RgpA inhibition by Kyt-1 on gingipain-triggered NETosis in murine bone marrow neutrophils was much stronger than in human neutrophils, as the inhibitor nearly completely blocked DNA release from mouse neutrophils (Fig 3C). This result was confirmed by the confocal microscopy using murine peritoneal neutrophils (S4 Fig). Notably, Kyt-1 and Kyt-36 acted selectively on gingipain activity, since they had no effect on NET generation induced by PMA or *S*. *aureus* (S5 Fig). Taken together, these data demonstrate that *P*. *gingivalis*-induced NETosis is at least partially dependent on the proteolytic activity of the Arg-X gingipains.

### NOX-dependent NET generation by gingipains

**Fig 4:**
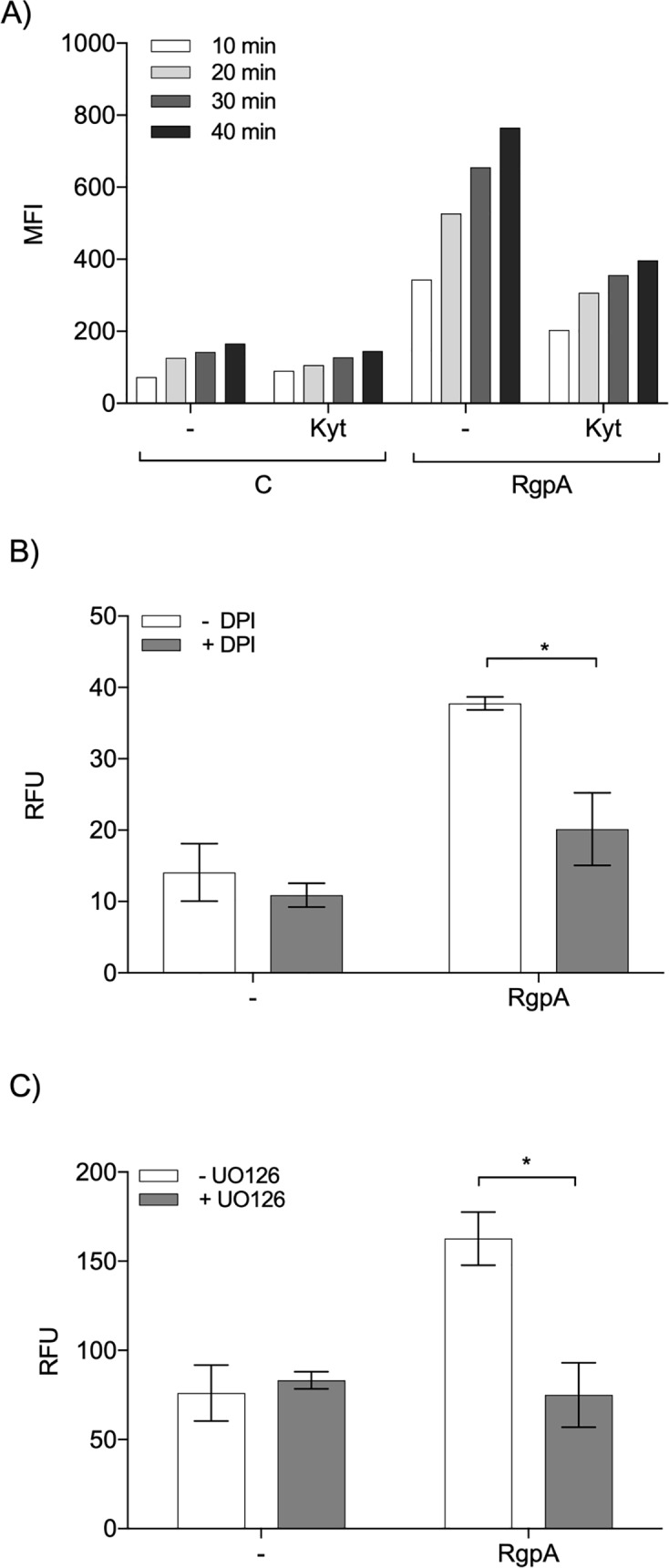
The signal transduction pathway triggered by gingipains. (A) Induction of respiratory burst by active and inactive (Kyt-treated) gingipains. Neutrophils were pretreated with 20 μM DCFH-DA for 10 min, then 50 nM RgpA was added after pretreatment with Kyt-1 or a vehicle control (1 μM). Data represent the mean fluorescence intensity (MFI) of the DCF-positive cells measured at 10, 20, 30, and 40 min after stimulation with RgpA. A representative result from three independent experiments is shown. (B, C) Cells were pretreated with 5 μM DPI (NADPH inhibitor) (B) or 10 μM UO126 (ERK inhibitor) (C) for 30 min. Then, neutrophils were exposed to 10 nM (B) or 50 nM (C) RgpA for 4 h. The level of NETs was determined by QPG. Statistical significance was evaluated by two-way ANOVA, followed by Bonferroni’s multiple comparisons posttest. Mean data (± SEM) from two independent experiments are shown. *P < 0.05; ns, non-significant.

NOX-mediated generation of ROS is one of the key pathways underlying NETosis [29]. Therefore, we examined whether the oxidative burst in neutrophils is crucial for gingipain-triggered NET formation. As determined by flow cytometry, incubation of neutrophils with 50 nM RgpA resulted in a time-dependent increase in ROS levels (Fig 4A). Conversely, the inhibitor-treated RgpA also induced respiratory burst, but at a clearly lower level, indicating that oxidative burst is largely dependent on the proteolytic activity of the gingipain (Fig 4A). Moreover, preincubation of neutrophils with 5 μM DPI, a selective inhibitor of NOX-dependent generation of NETs [33], followed by stimulation with active RgpA reduced NET formation to about half the levels seen in untreated cells (Fig 4B).

To further evaluate the importance of ROS in RgpA-induced NET formation, neutrophils were preincubated with a specific inhibitor (UO126) of the ERK, the main kinase in the NOX pathway [34], and NETosis was then induced with RgpA. Treatment with the ERK inhibitor led to a significant reduction in NET generation in response to RgpA (by about 50%) (Fig 4C). Taken together, these data indicate that the mechanism of NET generation by proteolytically active gingipain depends on NOX activation and ERK-dependent signaling.

### The mechanism of NET generation by gingipains

**Fig 5:**
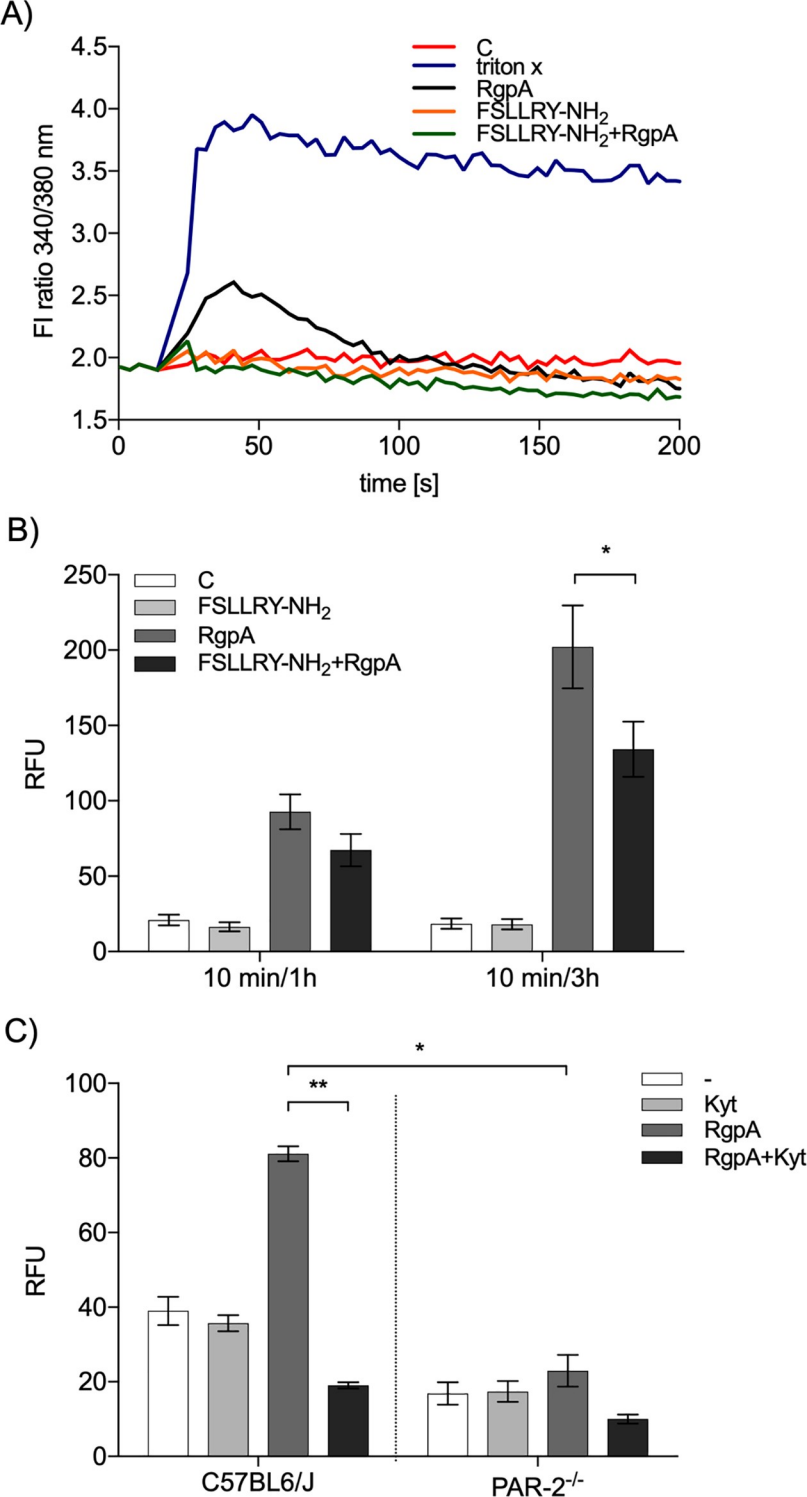
Activation of PAR-2 in NETosis induced by gingipains. (A) Neutrophils were loaded with Fura-2, then exposed to 100 μM FSLLRY-NH_2_, followed by 200 nM RgpA. Triton-X was used as a positive control for cellular calcium influx. The cytoplasmic concentration of calcium in a representative experiment is shown. (B) Neutrophils were stimulated for 1 and 3 h with active or inactive RgpA (50 nM) after preincubation with 100 μM FSLLRY-NH_2_ for 10 min. Statistical significance was evaluated by one-way ANOVA, followed by Bonferroni’s multiple comparisons posttest. Mean data (± SEM) from three independent experiments are shown. *P < 0.05. (C) Peritoneal neutrophils from WT C57BL6/J and PAR-2^-/-^ mice were stimulated for 4 h with 50 nM RgpA with or without pretreatment with Kyt-1 (1 μM). The level of extracellular DNA was estimated by QPG. Statistical significance was evaluated by two-way ANOVA, followed by Bonferroni’s multiple comparisons posttest. Mean data (± SEM) from one experiment using neutrophils from six mice per group are shown. *P < 0.05, and **P < 0.01.

The observation that NETosis is dependent on the proteolytic activity of gingipains implies the proteolytic cleavage of a protein(s) on the surface of neutrophils. Since it is well documented that Rgps activate signaling pathways involving protein G-coupled protease-activated receptors (PAR-1–4) in different cell types [35–38], we focused on PAR-2, which is the most abundant PAR on human and murine neutrophils. The extracellular N-terminus of PAR-2 is a promiscuous target for multiple proteases that cleave it at different sites. They either activate PAR signaling by unmasking an N-terminal self-activating tethered ligand or disarm the receptor by cleaving it downstream from the tethered ligand sequence [39, 40]. Since the ability of RgpB to trigger NETosis was significantly lower than that of RgpA (Fig 2B), in follow up experiments we focused only on RgpA. Initially we confirmed that RgpA cleaves a synthetic substrate bearing a PAR-2 sequential motif with the canonical activation cleavage site (…SKGR^36^ / SLIGRL…, where slash indicates the site where peptide bond cleavage occurs) (S6 Fig) in keeping with previously published data [35]. Then, to examine the role of PAR-2 engagement in NETosis, we showed that low molecular mass antagonists of PAR-2 efficiently blocked intracellular calcium mobilization induced by RgpA (Fig 5A) and significantly, reduced NETosis (Fig 5B). Therefore, as additional validation of the role of PAR-2 in NET formation, the response of mouse neutrophils isolated from WT and PAR-2-deficient mice to RgpA was compared. As shown in Fig 5C, in contrast to WT cells, neutrophils lacking PAR-2 did not exhibit NET formation when treated with RgpA. Of note, as in human neutrophils, blocking RgpA activity eliminated its ability to stimulate NET formation in mouse neutrophils.

Taken together, these data clearly demonstrate a role for the PAR-2 signaling pathway in gingipain mediated NET formation. PAR-2 is apparently activated by cleavage of the extracellular N-terminus at a canonical site (Arg36↓Ser37), exposing a tethered ligand at the new N-terminal receptor sequence.

### Bactericidal activity of NETs induced by *P*. *gingivalis* proteases

**Fig 6:**
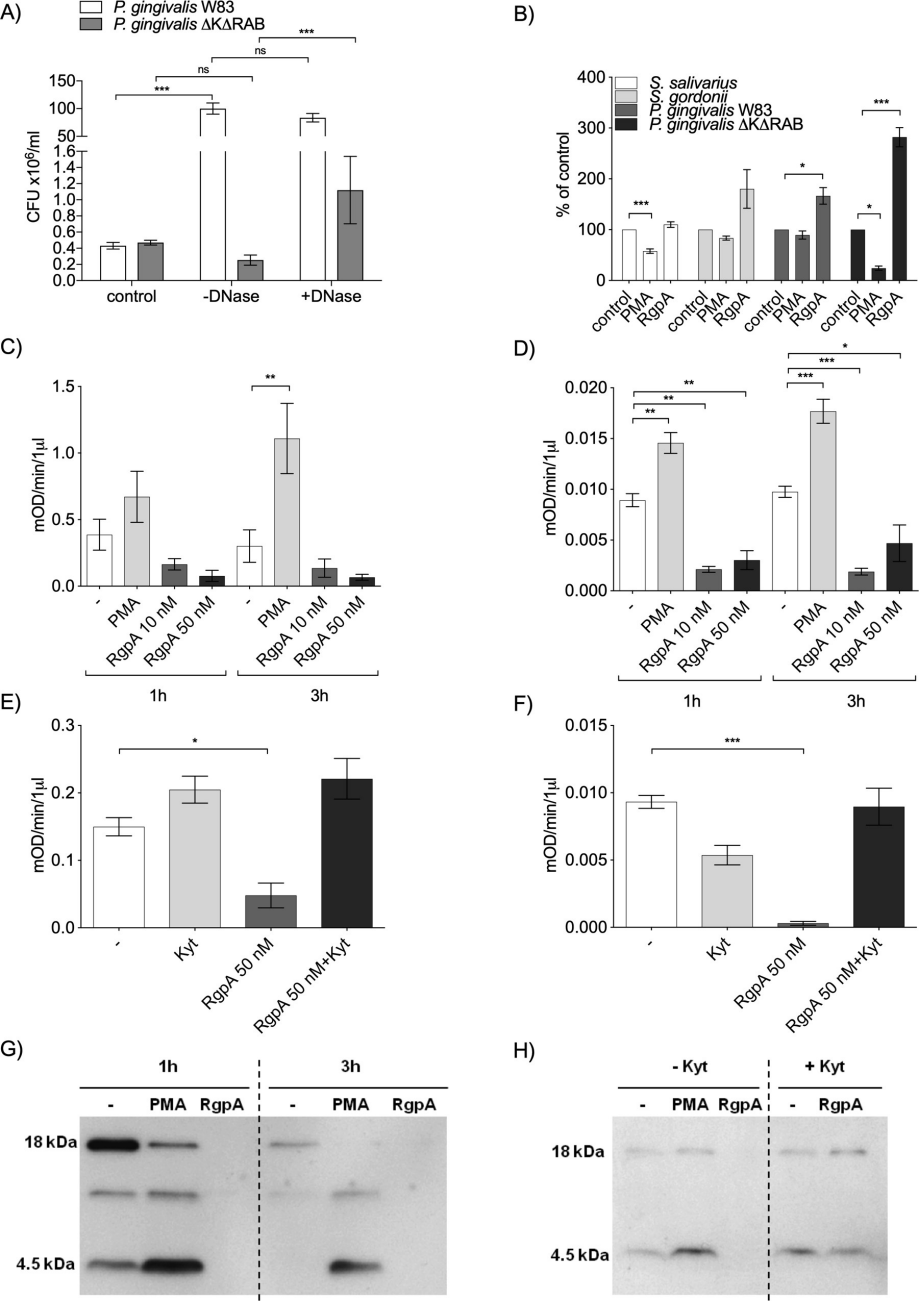
Bactericidal activity of NETs induced by gingipains. (A) Neutrophils in serum-free DMEM were infected with *P*. *gingivalis* W83 and/or ΔKΔRAB (MOI 1:10) in the presence or absence of DNase I. In parallel, bacteria were inoculated into the same medium but without neutrophils. After 3 h incubation mixtures of bacteria with neutrophils (with or without DNase) or bacteria alone in medium (control) were plated and CFUs were determined. (B) Selected bacterial species (MOI 1:5) were added to PMA (25 nM)- or RgpA (50 nM)-derived NETs or serum-free DMEM alone. After 2 h of incubation, bacteria were plated, and CFUs were determined. For each bacterium CFU in the control (bacteria in medium) was taken as 100% and bacterial survival after exposure to differently induced NETs was calculated as percent of the appropriate control. (A, B) Statistical significance was evaluated by one-way ANOVA, followed by Bonferroni’s multiple comparisons posttest. Mean data (± SEM) from three separate experiments are shown. *P < 0.05 and ***P < 0.001; ns, non-significant. (C–F) Enzymatic activity of human NE (C, E) and cat G (D, F) in NETs generated by PMA (25 nM) and/or RgpA in the presence or absence of a specific protease inhibitor (1 μM Kyt-1). Statistical significance was evaluated by unpaired t-test. Data represent the mean ± SEM of three independent experiments. *P < 0.05, **P < 0.01, and ***P < 0.001. (G, H) The presence of LL-37 within NETs generated by PMA (25 nM) or RgpA (50 nM) was visualized by immunoblot analysis at 1 h (G, H) and 3 h post-stimulation (G), in the presence of 1 μM Kyt-1 (H). A representative immunoblot from three separate experiments using neutrophils derived from different donors is shown.

NETosis is an important function of the immune defense system executed by neutrophils. Therefore, the bactericidal activity of NETs generated in response to *P*. *gingivalis* infection was analyzed. First, the killing efficiency of NETs induced by the WT strain of bacteria (W83) was compared to the killing induced by the gingipain-deficient strain (ΔKΔRAB). As a control we used the same amount of bacteria but incubated in culture media without neutrophils. In contrast to the gingipain-deficient strain (ΔKΔRAB), *P*. *gingivalis* expressing gingipains survived and even proliferated in the presence of NETs (Fig 6A). Degradation of NETs with DNase reversed the bactericidal activity against the gingipain-deficient strain (ΔKΔRAB) and even promoted bacterial growth. By contrast, proliferation of the WT strain occurred independently of DNA integrity (Fig 6A). To further investigate the lack of bactericidal activity of NETs induced by *P*. *gingivalis*, the efficiency of NETs induced by RgpA and PMA was compared. For this purpose, NETs induced by RgpA and PMA were inoculated with *P*. *gingivalis* WT and ΔKΔRAB, *S*. *salivarius*, and *S*. *gordonii*. PMA-triggered NETs significantly reduced the numbers of *S*. *salivarius* and *P*. *gingivalis* ΔKΔRAB, but had no effect on WT *P*. *gingivalis* and *S*. *gordonii*. Notably, RgpA-induced NETs showed no bactericidal activity but instead supported the growth of *S*. *gordonii* and both strains of *P*. *gingivalis* tested (Fig 6B). Collectively, these results suggest that, in the environment of *P*. *gingivalis*-infected periodontal pockets, NETs induced by Rgps may promote the proliferation of dysbiotic bacterial flora rather than exerting bactericidal activity.

### Modification of bactericidal components of NETs by gingipains

NETs induced by WT *P*. *gingivalis* show strongly reduced antibacterial activity, which is dependent on decoration of DNA strands with bactericidal peptides (LL-37, defensins) and proteins (cathepsin G (cat G), neutrophil elastase (NE), protease 3, pentraxin 3, lactoferrin, and others). Proteolytic inactivation of bactericidal components of NETs induced by gingipains may underlie the lack of bactericidal activity of these NETs. Therefore, we used SDS-PAGE to compare the proteins associated with DNA in NETs induced by WT *P*. *gingivalis* and the gingipain-deficient mutant at different MOIs. A strong difference in the protein band pattern was observed, suggesting extensive proteolysis in the NETs triggered by WT *P*. *gingivalis* (S7 Fig). Next, we focused on major bactericidal components in NETs, such as NE, cat G, and peptide LL-37. NE and cat G activity were compared in NETs induced by gingipains and PMA. While NE and cat G activity increased in a time-dependent manner in NETs triggered by PMA (Fig 6C and 6D), neutrophil serine protease activity remained below the background level in gingipain-induced NETs (Fig 6C and 6D). Remarkably, NE and cat G activity could be partially rescued by treatment of NETs with Kyt-1, a specific inhibitor of Arg-X gingipains, confirming NE and cat G degradation by RgpA (Fig 6E and 6F).

In NETosis, the LL-37 cathelicidin, which is released from an 18 kDa precursor (hCAP18) and binds to DNA, constitutes the most potent antibacterial component of the NET structure [41, 42]. Therefore, levels of LL-37 and its precursor protein were evaluated in NETs induced by PMA and RgpA. In PMA-induced NETs, both hCAP18 and the released LL-37 peptide (4.5 kDa) were observed. By contrast, in NETs triggered by RgpA, neither the precursor protein nor LL-37 was detected (Fig 6G). Again, as in the case of NE and cat G, treatment of NETs with the gingipain inhibitor Kyt-1 prevented the degradation of LL-37 (Fig 6H).

Collectively, these data clearly indicate proteolytic inactivation of bactericidal components of NETs by gingipains, which may explain the lack of antibacterial activity of NETs induced by *P*. *gingivalis*.

## Discussion

Impaired NET formation increases the susceptibility of the host to infection [43]. Although NETs promote elimination of pathogens, uncontrolled generation of neutrophil traps may intensify the inflammatory response [44, 45]. In the latter context, NETs are thought to be a mechanism underlying chronic bacterial diseases, including periodontitis [46, 47], where NETs have been documented in purulent periodontal exudates from patients [23] (Fig 1). Despite the presence of NETs in GCF, little is known about the mechanism of NET formation in periodontitis and their role in the pathogenesis of this chronic disease. Therefore, we evaluated the role of *P*. *gingivalis* and its main virulence factors, gingipains, in the process of NETosis. We showed that *P*. *gingivalis* generates extracellular NETs in human neutrophils isolated from the peripheral blood of healthy donors in a predominantly gingipain-dependent manner (Fig 1). An alternative mechanism of *P*. *gingivalis*-induced NETosis must also exist, since *P*. *gingivalis* deficient in all three gingipains (ΔKΔRAB) was still capable of inducing NET formation. This corroborates the findings by other groups that gingipain mutants (both Kgp and RgpA/RgpB) were able to trigger NETosis [48]. Although, our studies excluded the direct involvement of LPS and FimA, it is still likely that NETosis induced in response to gingipain-null bacterial cells is the result of neutrophils responding to the simultaneous recognition of several virulence factors. Alternatively a recently reported no canonical inflammasome signaling pathway that triggers gasdermin D-dependent neutrophil death may be considered as responsible for NETosis stimulated by engulfed gingipain-null *P*. *gingivalis* escaping a phagosome into the cytoplasm [49–51].

Using purified gingipains and isolated OMVs from WT *P*. *gingivalis*, we confirmed that NETosis was selectively triggered by arginine-specific gingipains in a proteolysis-dependent manner. Gingipains acting on neutrophils have also been shown to cause recognition and engulfment of healthy cells by macrophages [52]. Although NETosis was not studied in this work, it is likely that the observed effect reflects the clearance of NETs by macrophages [53].

A detailed analysis of the mechanism of NET generation by RgpA demonstrated the involvement of the NOX pathway, ROS release, and ERK kinase signaling (Fig 4). Moreover, the observed process bears the marks of suicidal NETosis, which is characterized by a long duration of NET generation (about 4 h) and is associated with damage of the neutrophil membrane and cell death [29]. Until now, the initial event that triggers the NOX-dependent pathway, leading to NETosis, remained undetermined. A large spectrum of cell surface receptors and proteins are proteolytic targets of gingipains [54]. We focused on the role of PAR receptors, which are activated by proteolytic truncation of their extracellular domain. The newly generated N-terminal is recognized as a specific ligand that reversibly activates cell signaling. Our data revealed, for the first time, that PAR-2 is involved in the process of NET induction in response to gingipains. This contributes to a growing body of evidence suggesting that signaling initiated by activation of PAR-2 on neutrophils and epithelial cells plays a detrimental role in the pathobiology of periodontitis [35, 55, 56]. Therefore, inhibition of PAR signaling should be considered as a novel therapeutic approach in pathological conditions where excessive NETosis is observed. We must keep in mind, however, that gingipains can penetrate the cell membrane [57] and may alter intracellular necroptotic signaling through RIPK1 degradation [58, 59].

Interestingly, despite the fact that the catalytic domains of RgpA and RgpB are nearly identical, RgpA was a much better NET inducer than RgpB at the equimolar active site concentration (Fig 2). This difference may be due to the presence of hemagglutinin/adhesion (HA) domains in the RgpA molecule, which are absent from RgpB [60]. This suggestion is supported by the observation that blocking the catalytic site of RgpA with a specific inhibitor only reduced the ability of the active enzyme to induce NETosis in human neutrophils by approximately 50% (Fig 3A). Moreover, Kyt-1 was a much stronger inhibitor of NETosis triggered by RgpB than by RgpA. Therefore, we postulate that the role of gingipains in NET formation is not limited to their enzymatic activity but depends also on their interactions with as-yet-unknown receptors in the cell membrane. This hypothesis is consistent with our observations as well as those of Fitzpatrick *et al*., who showed that proteolytically inhibited forms of RgpA and Kgp efficiently activate macrophages, leading to the release of pro-inflammatory cytokines [61, 62]. As the mechanism, the authors suggested that the HA domains of those enzymes stimulate TLRs, including TLR2 and TLR7 [61].

Several pathogens have evolved mechanisms that efficiently protect them against elimination by NETs. In addition to secretion of extracellular DNases, the most common defense against trapping in NETs [63, 64], other mechanisms include expression of the M1 protein by *S*. *pyogenes* and assembly of a protective surface lipophosphoglycans layer by *Leishmania donovani* [6, 65]. In this study, we describe yet another mechanism for evading the antimicrobial activity of NETs. This novel strategy depends on the proteolytic activity of gingipains. Due to their specificity for Arg-X or Lys-X peptide bonds, gingipains can very efficiently degrade cationic antibacterial components anchored to the chromatin backbone, including LL-37 [16], defensins [66], elastase, and cat G. By using gingipains to inactivate neutrophil serine proteases *P*. *gingivalis* hijacks a host strategy for disarming pathogens by proteolytic degradation of their virulence factors in the DNA meshwork of NETs.

The pathological significance of this new mechanism of virulence, which relies on the formation of NETs devoid of bactericidal activity, is the generation of an environment that favors pathogen proliferation (Fig 6). *P*. *gingivalis* is a fastidious asaccharolytic microbe that can flourish in an environment rich in peptides and growth factors released by dying neutrophils and generated by degradation of proteins decorating the DNA fibers of NETs [67, 68]. Moreover, the growth-promoting environment created by *P*. *gingivalis* in periodontal pockets, where NETs are copious [24], is shared with the other dysbiotic inhabitants of subgingival plaque [69]. Many of these species are likely susceptible to killing by antibacterial peptides and proteins/enzymes, but co-habitation with *P*. *gingivalis* protects them against the bactericidal activity of NETs, allowing them to proliferate and colonize the inflamed tissue. This is likely a way that *P*. *gingivalis* maintains its status as a keystone pathogen in the subgingival community of microbes [70–72].

Altogether, our data indicate once again that gingipains are effective weapons through which bacterial invaders hijack the primary function of innate immunity for their own benefit. Induction and regulation of NETs plays a large role in promoting the process of dysbiosis, by influencing the viability of commensal flora and supporting the survival and growth of pathobionts. Moreover, the expression of intracellular self-antigens in gingipain-induced NETs contributes to the creation of a favorable environment for the development of autoimmune disorders. For this reason, elucidation of the role of *P*. *gingivalis* in the development of NETs and the precise determination of the bacterial antigens that mediate this process is of great clinical significance.

In conclusion, our studies revealed a likely mechanism of triggering NET formation that is dependent on activation of PAR-2 by *P*. *gingivalis*-derived proteases. Furthermore, we also demonstrated a novel role for proteases as bacterial virulence factors antagonizing the antibacterial activity of NETosis. Our results suggest that generation of NETs in the periodontium enhances inflammation and can be considered yet another virulence strategy used by *P*. *gingivalis*. Importantly, the presentation of intracellular self-antigens modified by gingipains may have immunological consequences, as the excessive presentation of cryptic antigens plays a role in the development of systemic diseases associated with periodontitis.

## Methods

### Human neutrophil isolation

Peripheral blood from de-identified human donors was obtained from the Red Cross (Krakow, Poland). Neutrophils were isolated from granulocyte-enriched fractions, which were harvested by centrifugation over a density gradient using a lymphocyte separation medium (Pan Biotech, Germany). Neutrophils and erythrocytes were collected as the high-density fraction and separated after 30 min of incubation with 1% polyvinyl alcohol (POCH, Poland). Neutrophils were collected from the upper layer, and after centrifugation (280 × g, 10 min), the residual erythrocytes were removed by lysis in water. Neutrophils were resuspended in serum-free DMEM without phenol red (Gibco/ThermoFisher Scientific, USA).

### Isolation of murine neutrophils from bone marrow

Bone marrow-derived neutrophils were isolated using previously published methods, with slight modifications [73, 74]. The femur and tibia were removed from 8-week-old C57BL6/J female mice. Bone marrow was extracted by flushing the bones with RPMI 1640 (Gibco/ThermoFisher Scientific) using a 25G needle and passed through a 70 μm cell strainer to obtain a single-cell suspension. After centrifugation (300 × g, 10 min), the erythrocytes were lysed using red blood cell lysis buffer (0.83% NH_4_Cl, 0.1% NaHCO_3_, 0.004% EDTA), and the bone marrow was centrifuged again (300 × g, 10 min). Collected cells were resuspended in fresh medium. Granulocytes were separated from mononuclear cells by centrifugation (500 × g, 10 min) over a discontinuous Percoll (Sigma-Aldrich, USA) density gradient consisting of 55%, 65%, and 75% Percoll in HBSS (Sigma-Aldrich). Mature neutrophils were recovered from the interphase between the 65% and 75% Percoll into serum-free DMEM without phenol red and centrifuged (280 × g, 10 min). Murine neutrophils were resuspended in serum-free DMEM without phenol red at a density of 0.1 × 10^6^/well and plated in 96-well plates. This method leads to a myeloid preparation consisting of approximately 90% neutrophils, based on cytology.

### Thioglycollate-induced peritonitis in mice

Peritonitis was induced in 6–8-week-old female wild-type (WT; PAR-2^+/+^) and PAR-2-deficient (PAR-2^-/-^) C57BL6/J mice. Mice were injected intraperitoneally with 1 ml of 4% sterile thioglycollate (Fluka, USA). Peritoneal exudates were collected after 3 h by washing the peritoneal cavity with 10 ml of ice-cold PBS (Gibco/ThermoFisher Scientific). The cells were centrifuged at 280 × g for 5 min, and after lysis of red blood cells, peritoneal neutrophils were counted and subjected to flow cytometry with a FITC-conjugated rat anti-mouse Ly-G6 antibody (BD Biosciences, USA).

### Bacterial strains and cultures

*P*. *gingivalis* WT strains W83, W50, A7436, and HG66, and the gingipain-null isogenic mutant W83ΔKΔRAB, were grown on blood agar plates (5% sheep blood) supplemented with 1 μg/ml tetracycline for the gingipains mutant or in Schaedler broth liquid medium (BTL, Poland) supplemented with hemin (1 mg/ml; Sigma-Aldrich), menadione (0.5 mg/ml; ICN Biomedicals, USA), and L-cysteine (50 mg/ml; BioShop, Canada). *P*. *gingivalis* WT strains ATCC 33277 and 381, and the gingipain-null isogenic mutant KDP 136 on the ATCC 33277 background [75], were grown on blood agar plates (5% sheep blood) supplemented with 1 μg/ml tetracycline, 5 μg/ml erythromycin, and 20 μg/ml chloramphenicol for the gingipains mutant or in Brain Heart Infusion broth liquid medium (BD Biosciences) supplemented with hemin (1 mg/ml; Sigma-Aldrich) and menadione (0.5 mg/ml; ICN Biomedicals). All strains were grown at 37°C under anaerobic conditions (90% N_2_, 5% CO_2_, 5% H_2_). Bacteria from an overnight culture were centrifuged (5,000 × g, 5 min, 4°C), washed twice with PBS, and resuspended in PBS to an optical density at 600 nm (OD_600_) of 1.0, which corresponds to 1 × 10^9^ colony-forming units (CFUs) ml^-1^.

*Streptococcus salivarius* ATCC 7073 and *Streptococcus gordonii* ATCC 10558 were inoculated from blood agar plates (5% sheep blood) into 20 ml of Tryptic Soy Broth (Sigma-Aldrich) and grown overnight to the stationary growth phase at 37°C under constant rotation (180 rpm). Prior to each inoculation, the bacterial cells were collected by centrifugation (5,000 × g, 5 min, 4°C), washed twice with PBS, and resuspended in PBS to the desired OD_600_.

### Isolation of *P*. *gingivalis* OMVs

*P*. *gingivalis* (OD_600_ = 1) grown in liquid Schaedler broth were sonicated in a water bath to facilitate the release of OMVs from the bacterial surface. The bacterial suspension was then centrifuged (10,000 × g, 20 min, 4°C), and OMVs in the supernatant were collected by ultracentrifugation (150,000 × g, 1 h, 4°C). The OMV pellet was resuspended in 20 mM BisTris, 150 mM NaCl, and 5 mM CaCl_2_ (pH 6.8). The amount of protein in isolated OMVs was determined by bicinchoninic acid (BCA) assay.

### Proteolytic enzymes

Arg-X gingipains (RgpA and RgpB) and the Lys-X gingipain (Kgp) were purified from spent growth media of *P*. *gingivalis* HG66, as described previously [76, 77]. The concentrations of active Rgp and Kgp gingipains were determined by active site titration using the gingipain-specific inhibitors Kyt-1 and Kyt-36, respectively (Peptide Institute, Japan) [32]. The purified enzymes were activated by 15 min incubation at 37°C in 100 mM Tris-HCl, 150 mM NaCl, 5 mM CaCl_2_, and 20 mM cysteine (pH 7.5), and then diluted to the required concentrations in culture medium supplemented with 10 mM cysteine. Gingipain activity was inhibited by incubating cells with Kyt-1 and/or Kyt-36 (1 μM) for 15 min at 37°C. The efficiency of enzyme inhibition was verified using L-BA*p*NA (Sigma-Aldrich) as a substrate for Arg-X gingipains and Tos-GPK-*p*NA (Sigma-Aldrich) for the Lys-X gingipain.

### Induction and quantification of NETs

In the majority of experiments, human neutrophils were seeded at 2 × 10^6^/well in 0.01 mg/ml poly-L-lysine (Sigma-Aldrich)-coated 24-well plates and centrifuged (200 × g, 5 min) to allow cells to adhere to the plates. Then, neutrophils were stimulated at 37°C with the following: (*i*) different strains of *P*. *gingivalis* at a multiplicity of infection (MOI) of 1:5, 1:50, or 1:100; (*ii*) OMVs isolated from W83 or ΔKΔRAB at a concentration of 25 μg/ml that resembles MOI 1:100; (*iii*) purified gingipains, separately or together at a final concentration of 10 or 50 nM each, in the presence or absence of Kyt-1 or Kyt-36 (1 μM); (*iv*) *P*. *gingivalis* LPS and fimbriae A (FimA) at concentrations from 0.1 to 1 μg/ml; (*v*) phorbol ester (PMA; Sigma-Aldrich) at a concentration of 25 nM.

For inhibition of nicotinamide adenine dinucleotide phosphate (NADPH) oxidase (NOX)-dependent ROS production, neutrophils were pretreated with 5 μM diphenylene iodonium (DPI; Sigma-Aldrich) for 30 min prior to treatment with RgpA (50 nM) for 4 h. To investigate selected signaling pathways during RgpA-triggered NETosis, neutrophils (0.1 × 10^6^/well in 0.01 mg/ml poly-L-lysine-coated 96-well plates) were pretreated for 30 min with 10 μM of an extracellular signal-regulated kinase (ERK) inhibitor (UO126; Cell Signaling Technology, USA) before incubation with 50 nM RgpA for 4 h.

To determine the role of PAR-2 in NET formation by gingipains, neutrophils (2 × 10^6^/well) were pretreated for 10 min with 100 μM of a PAR-2 antagonist peptide (FSLLRY-NH_2_; Tocris Bioscience) before adding active or inactive 50 nM RgpA for 1 or 3 h.

In the majority of experiments, neutrophils were incubated at 37°C under aerobic conditions (humidified atmosphere of 5% CO_2_). Certain experiments, as indicated in the Results section, were performed under anaerobic conditions (90% N_2_, 5% CO_2_, 5% H_2_). At the indicated time points, culture media from untreated (control) or treated neutrophils was collected and the amount of extracellular DNA was quantified using Quant-iT PicoGreen dsDNA Reagent (QPG; Invitrogen/ThermoFisher Scientific). QPG was diluted 1:200 in TE buffer [10 mM Tris, 1 mM EDTA (pH 7.5)], and 90 μl was mixed with 10 μl of supernatant containing the liberated extracellular DNA. The fluorescence was measured at an excitation wavelength of 480 nm and an emission wavelength of 520 nm. For NET degradation, supernatant collected from netting neutrophils was treated with 50 μg/ml DNase I (Roche, Switzerland). After 15 or 45 min of DNase I treatment, 2 mM EDTA was added to stop the reaction. The efficiency of DNA degradation in NETs was determined by comparison with non-DNase-treated samples.

### Confocal fluorescence microscopy

Neutrophils were plated at 5 × 10^5^ cells on poly-L-lysine-coated coverslips. After 30 min of incubation at 37°C, the cells were left untreated or stimulated with 10 nM gingipains for 4 h. Neutrophils were fixed with 3.7% formaldehyde for 10 min, washed three times with PBS, and blocked with 5% FBS, 1% BSA, 0.05% Tween, and 2 mM EDTA in PBS for 1 h. Cells were washed and treated with 0.1% saponin (Sigma-Aldrich) in PBS for 30 min. Cells were stained with the following antibodies in PBS containing 3% BSA and 0.1% saponin: rabbit anti-human neutrophil elastase (NE; Athens Research and Technology, USA) for 1 h, followed by APC-conjugated goat anti-rabbit IgG F(ab’)_2_ (Jackson ImmunoResearch Laboratories, USA) for 45 min. Cells were counterstained with 1 μg/ml Hoechst 33342 (Invitrogen/ThermoFisher Scientific), a DNA-intercalating dye. Preliminary images were taken with an Olympus Fluoview microscope. Robust, automated quantification of NETs was done using Atomic J software [78] in a manner similar to what has been previously described for MATLAB based quantification [79]. Briefly, quantification of NETs was performed by using two fluorescent channels. Images of Hoechst bound DNA (blue 488 laser) and extruded elastase (immunofluorescence, Red 638nm laser) were acquired using a Zeiss LSM 880 confocal laser scanning microscope. Total ‘NET area’ was determined as the overlapping area with merged DNA and elastase immunofluorescence. Similarly, DNA fluorescence was obtained from cells not undergoing NETosis (circular DNA from unstimulated cells, or cells with decondensed chromatin that does no co-localize with elastase) and described as samples non-containing NETs. Therefore, in the case of such samples the ‘NET area’ was not calculated. Quantification of NETs was done based on the percentage value of NET area with respect to the total area of an image. We consider such an approach more appropriate than calculation of a number of NETs per cell previously described by Brinkman *et al* using ImageJ [80], because some NETs were stretched on the entire image and it was impossible to determine to which cell it should be attributed. Controls, such as unstimulated cells (with no NET area) and positive controls (PMA or *Staphylococcus aureus* stimulated cells), were used to verify the NETs quantification using our approach (S8 Fig).

### Scanning electron microscopy (SEM)

GCF or neutrophils isolated from blood of healthy donors (5 × 10^5^ cells/coverslip) were seeded on poly-L-lysine-coated coverslips. Probes were fixed in 2.5% glutaraldehyde in 0.1 M sodium cacodylate buffer (pH 7.4). After fixation, the sections were washed in sodium cacodylate buffer and post-fixed in 1% osmium tetroxide. Next, samples were dehydrated in an alcohol series, dried, and sputtered with gold. Images were captured with a JSM5410 scanning electron microscope (JEOL) at the Institute of Zoology, Jagiellonian University, in Krakow, Poland or Jena University, in Germany.

### Respiratory burst of neutrophils

The induction of respiratory burst was measured by the oxidation of dichloro-dihydro-fluorescein diacetate (DCFH-DA; Sigma-Aldrich) to fluorescent DCF. Neutrophils (1 × 10^6^ cells/well) were resuspended in PBS and treated with 20 μM DCFH-DA at 37°C for 10 min. Neutrophils were left untreated or stimulated with Arg-X gingipains at a final concentration of 50 nM in the presence or absence of Kyt-1 (1 μM). The mean fluorescence intensity (MFI) was determined by flow cytometry 10, 20, 30, and 40 min after stimulation at excitation and emission wavelengths of 492–495 nm and 517–527 nm, respectively. Data were acquired on a FaCScan flow cytometer (Becton Dickinson; USA) and analyzed with CellQuest software.

### PAR-2 cleavage assays

A fluorescence-quenched peptide substrate with a sequence corresponding to a region spanning the cleavage site of PAR-2 was used (Anthraniloyl-Gly-Ser-Lys-Gly-Arg-Ser-Leu-Ile-Gly-3-Nitro-Tyr-Asp-amide). The substrate at a final concentration of 10 mM was incubated with 1 nM gingipains in 200 μl of buffer [100 mM Tris, 150 mM NaCl, 5 mM CaCl_2_, 0.05% Tween, 10 mM cysteine (pH 7.5)]. The buffer contained 5% dimethylformamide (DMF; Sigma-Aldrich). Enzymatic hydrolysis of the substrates was recorded at 37°C for 1 h, using a fluorescence microplate reader at excitation and emission wavelengths of 290 nm and 400 nm, respectively.

### Intracellular calcium measurement

The cytosolic Ca^2+^ concentration was measured in suspensions of 4 × 10^6^ neutrophils/ml in HBSS with NaHCO_3_ (Sigma-Aldrich). The cells were loaded with 5 μM Fura-2 (Invitrogen) for 30 min in the dark at 37°C. After centrifugation at 280 × g for 10 min at room temperature, cells were washed twice with HBSS with NaHCO_3_ and resuspended in HBSS with NaHCO_3_ at 2 × 10^6^ cells/ml. Finally, 0.2 × 10^6^ Fura-2-loaded cells were maintained at 37°C for fluorescence measurements using a Flex Station 3 multi-mode microplate reader (Molecular Devices) at excitation and emission wavelengths of 340/380 nm and 505 nm, respectively. Neutrophils were preincubated for 10 min with 100 μM PAR-2 antagonist, and after a stable baseline was established, 200 nM RgpA was added and the ratio of fluorescence at the two excitation wavelengths was measured. This value is proportional to the [Ca^2+^].

### NET-mediated bacterial killing

Neutrophils were seeded at 2 × 10^6^/well in 0.01 mg/ml poly-L-lysine-coated 24-well plates and incubated at 37°C with or without DNase I (100 units/ml) for 15 min prior to addition of *P*. *gingivalis* W83 or ΔKΔRAB at a MOI of 1:10 to form NETs. As a control, bacteria were incubated in serum-free DMEM without neutrophils. After 3 h, samples (100 μl each) were plated on blood agar plates and cultured anaerobically for 7 days at 37°C, after which time, visible colonies of *P*. *gingivalis* were counted to obtain the total viable cell numbers.

### Quantification of bactericidal activity of NETs

Neutrophils were seeded at 2 × 10^6^/well in 0.01 mg/ml poly-L-lysine-coated 24-well plates and stimulated for 4 h at 37°C with 25 nM PMA and/or 50 nM RgpA. Extruded NETs were collected and incubated with *S*. *salivarius* ATCC 7073, *S*. *gordonii* ATCC 10558, *P*. *gingivalis* W83, or ΔKΔRAB at a MOI of 1:5 (based on the number of neutrophils from which the NETs were collected). As a control, bacteria were incubated in supernatant from untreated neutrophils. After 2 h, bacterial survival was estimated by plating dilutions on blood agar plates and counting colonies to determine CFUs.

### SDS-PAGE and immunoblotting

NETs generated from neutrophils exposed to PMA and RgpA were collected, and equal amounts of protein were subjected to SDS-PAGE. After electrophoresis, the gel was stained with Coomassie brilliant blue G-250 (Serva, Germany) or electrotransferred onto PVDF membranes (Merck Millipore, USA) in 25 mM Tris and 0.2 M glycine (pH 8.3) supplemented with 20% methanol (60 V, 3 h, 4°C). Non-specific binding sites were blocked with 5% skim milk in TTBS (pH 7.5) for 4 h at room temperature, followed by overnight incubation at 4°C with a 1:500 dilution of anti-human LL-37/CAP-18 (Hycult Biotech, Netherlands) in TTBS containing 3% BSA. Membranes were washed extensively in TTBS and incubated with a 1:20,000 dilution of a sheep anti-mouse IgG-horseradish peroxidase (HRP) secondary antibody (Sigma-Aldrich) for 2 h in TTBS containing 3% BSA. Membranes were washed (5 × 5 min) in TTBS, and blots were developed using enhanced chemiluminescence (ECL) (ThermoFisher Scientific).

### Enzymatic activity assays

NETs generated in response to PMA or RgpA were collected, and the activities of neutrophil serine proteases were measured using specific substrates. NE activity was assayed using N-methoxysuccinyl-Ala-Ala-Pro-Val-p-nitroanilide (Sigma-Aldrich) as the substrate, while cat G activity was assayed using N-succinyl-Ala-Ala-Pro-Phe-p-nitroanilide (Sigma-Aldrich) as the substrate. The substrate [1 mM; in 100 μl of 50 mM Tris-HCl (pH 7.5)] was mixed with 100 μl of supernatant from the netting and control neutrophils, and the rate of substrate hydrolysis was measured as the increase in the optical density at 450 nm (OD_405_) after incubation for 30 min at 37°C.

### Statistical analyses

All experiments were performed in at least triplicate, and the results are expressed as the mean ± SEM. Statistical comparisons were performed with Prism 5.0 software (GraphPad), using two-tailed Student t-tests or one- or two-way factorial analyses of variance (ANOVA) followed by Bonferroni post-tests. Differences were considered significant when P < 0.05.

### Ethics statement

Gingival crevicular fluid was obtained from *P*. *gingivalis*—positive periodontitis patients. The ethical committee of Jena University, Germany, approved collection of GCF (2375-08/08). All volunteers were informed about the study and signed an informed consent prior to participation in the study. Human blood for PMNs isolation was purchased from Red Cross, Krakow, Poland. The Red Cross de-identified blood materials as appropriate for the confidentiality assurance of human subjects. Thus, this study adheres to appropriate exclusions from the approval of human subjects. All procedures performed using animals were approved by the local Institutional Animal Experimentation Ethics Committee (2nd Local Institutional Animal Care and Use Committee, permission numbers: 164/2013 and 191/2017) according to the national regulations (directive 2010/63/EU of the European Parliament).

## Supporting information

S1 Fig The level of NETs induced by different *P*. *gingivalis* strains. Neutrophils were stimulated with different strains of *P*. *gingivalis* (W83, W50, A7436, HG66, ATCC 33277, or 381) at MOIs of 1:5, 1:50, and 1:100 for 1 h. The level of NETs was determined by QPG. Mean data (± SEM) from a single experiment are shown.
(TIFF)

S2 Fig The role of gingipains in *P*. *gingivalis*-induced NET formation. (A) Comparison of *P*. *gingivalis*-mediated NET generation in aerobic and anaerobic conditions. Neutrophils were stimulated with *P*. *gingivalis* W83 and ΔKΔRAB at a MOI of 1:100 in aerobic or anaerobic conditions for 1 h. The level of NETs was determined by QPG. Data are the mean (± SEM) from a representative experiment. (B) Neutrophils were stimulated with WT *P*. *gingivalis* (ATCC 33277) and its isogenic mutant devoid of gingipain expression (KDP 136) at MOIs of 1:5, 1:50, and 1:100 for 1 h. The level of NETs was determined by QPG. Statistical significance was evaluated by two-way ANOVA, followed by Bonferroni’s multiple comparisons posttest. Mean data (± SEM) from a representative experiment are shown. ***P < 0.001.
(TIFF)

S3 Fig The formation of NETs by purified gingipains. (A) Neutrophils were stimulated with an equimolar mixture of all three gingipains (RgpA, RgpB, and Kgp, each at 10 nM) for 4 h. NET structures were visualized by SEM. (B) For confocal laser scanning microscopy, DNA was stained with Hoechst 33342 (blue), and human neutrophil elastase (HNE) was stained with an APC-labeled antibody (red). Bars represent 20 μm. Quantitative analysis of NETs images was performed by merging blue and red channels (merge/contours). Percentage of the NET area in relation to the area of an image is presented as mean data (± SEM) from three independent images. n.d.- not detected NETs.
(TIFF)

S4 Fig Visualization of NETs structures induced by RgpA. For confocal laser scanning microscopy neutrophils isolated from mouse peritoneal cavity were stimulated with 100 nM RgpA in the presence or absence of Kyt-1 at a final concentration of 1 μM. DNA is shown in blue (Hoechst 33342) and human neutrophil elastase (HNE) expression is shown in red. Bars represent 20 μm.
(TIFF)

S5 Fig The influence of Kyt-1 and Kyt-36 on NETs induction. Human peripheral blood neutrophils were stimulated for 1h and 4 h with 25 nM PMA and *S*. *aureus* at MOIs of 1:5, 1:25 with or without pretreatment with Kyt-1 (1 μM). The level of NETs was determined by QPG. Mean data (± SEM) from a single experiment are shown.
(TIFF)

S6 Fig Activation of PAR-2 fluorescence peptide by RgpA. PAR fluorescence-quenched peptide (10 mM) were activated by 1 nM RgpA. The cleavage of PAR-specific sequences was estimated by fluorimetry and compared to the fluorescence background measured for the probe without RgpA. The canonical cleavage site is presented on the figure. Statistical significance was evaluated by unpaired t-test. Mean data (± SEM) from two independent experiments are shown. ***P < 0.001.
(TIFF)

S7 Fig Gingipains modified the NET protein profile. W83- and ΔKΔRAB-induced NETs (MOI 1:50 and 1:100) were collected 1 h after infection of neutrophils. Samples were separated by SDS-PAGE. A representative gel from one experiment is shown.
(TIFF)

S8 Fig Quantification of NETs formation induced by 25 nM PMA and *S*. *aureus* at a MOI 1:5. (A) For confocal laser scanning microscopy, DNA was stained with Hoechst 33342 (blue), and human neutrophil elastase (HNE) was stained with an APC-labeled antibody (red). Bars represent 20 μm. A representative quantitative analysis of NETs images by merging blue and red channels (merge/contours). (B) Percentage of the NET area in relation to the area of an image. Mean data (± SEM) from three independent images. n.d.–NETs not detected.
(TIFF)
