# Supplementary figures and images for: Triggering NETosis via protease-activated receptor (PAR)-2 signaling as a mechanism of hijacking neutrophils function for pathogen benefits
Source: PLoS Pathog. 2019 May 20;15(5):e1007773. doi: 10.1371/journal.ppat.1007773 (PMC6544335; doi:10.1371/journal.ppat.1007773)

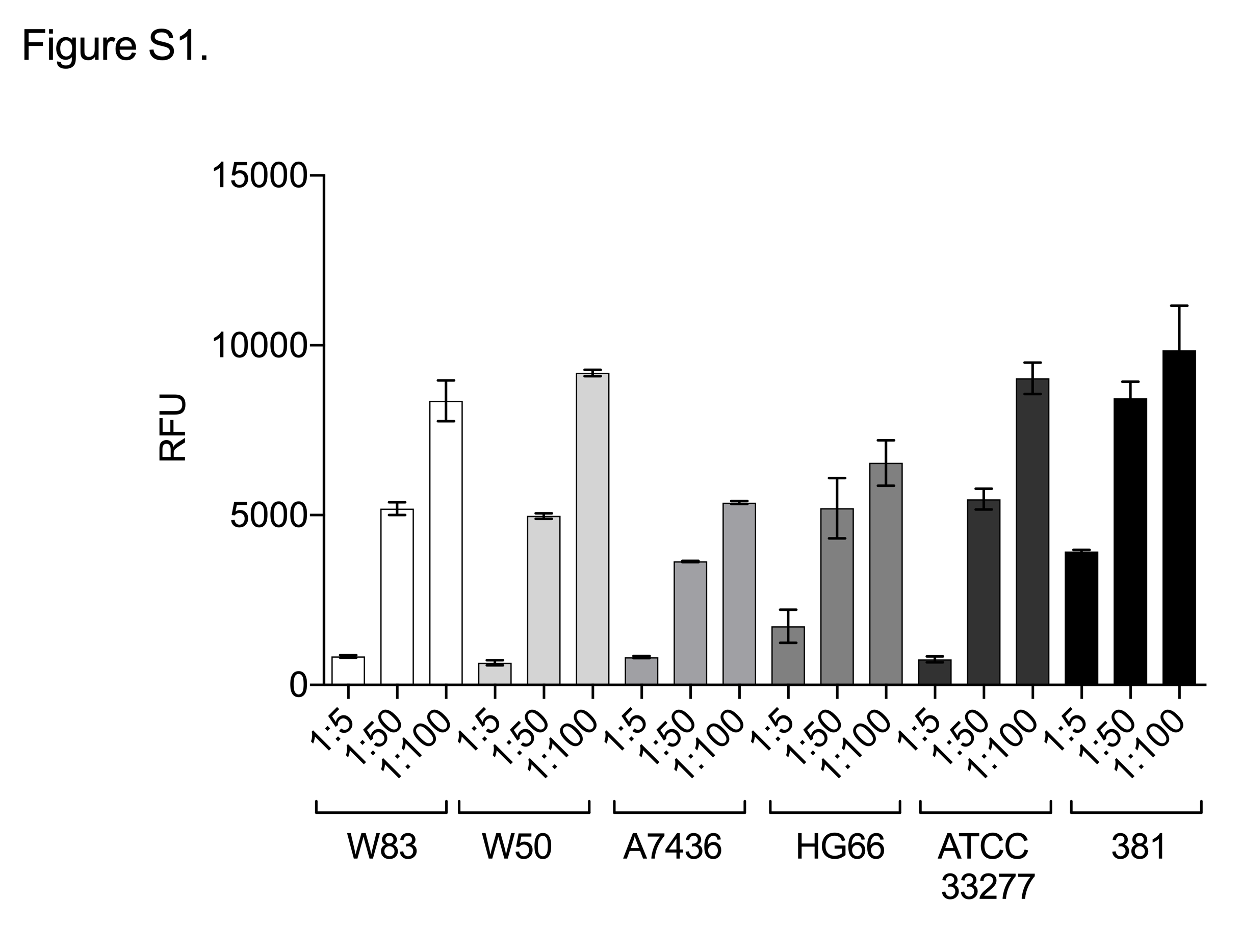

Supplement: S1 Fig — Neutrophils were stimulated with different strains of P. gingivalis (W83, W50, A7436, HG66, ATCC 33277, or 381) at MOIs of 1:5, 1:50, and 1:100 for 1 h. The level of NETs was determined by QPG. Mean data (± SEM) from a single experiment are shown. (TIFF) [file ppat.1007773.s001.tiff]

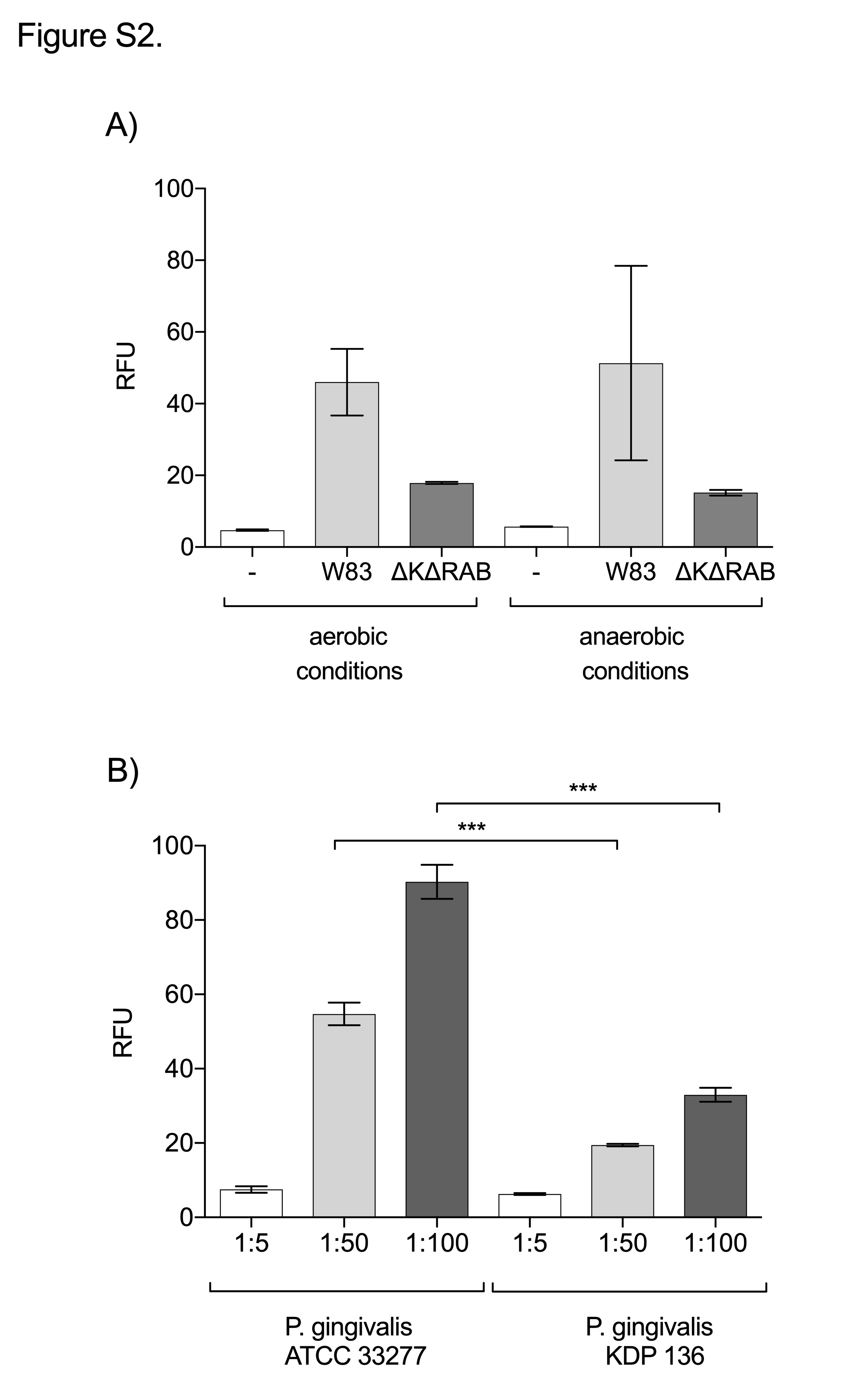

Supplement: S2 Fig — (A) Comparison of P. gingivalis-mediated NET generation in aerobic and anaerobic conditions. Neutrophils were stimulated with P. gingivalis W83 and ΔKΔRAB at a MOI of 1:100 in aerobic or anaerobic conditions for 1 h. The level of NETs was determined by QPG. Data are the mean (± SEM) from a representative experiment. (B) Neutrophils were stimulated with WT P. gingivalis (ATCC 33277) and its isogenic mutant devoid of gingipain expression (KDP 136) at MOIs of 1:5, 1:50, and 1:100 for 1 h. The level of NETs was determined by QPG. Statistical significance was evaluated by two-way ANOVA, followed by Bonferroni’s multiple comparisons posttest. Mean data (± SEM) from a representative experiment are shown. ***P < 0.001. (TIFF) [file ppat.1007773.s002.tiff]

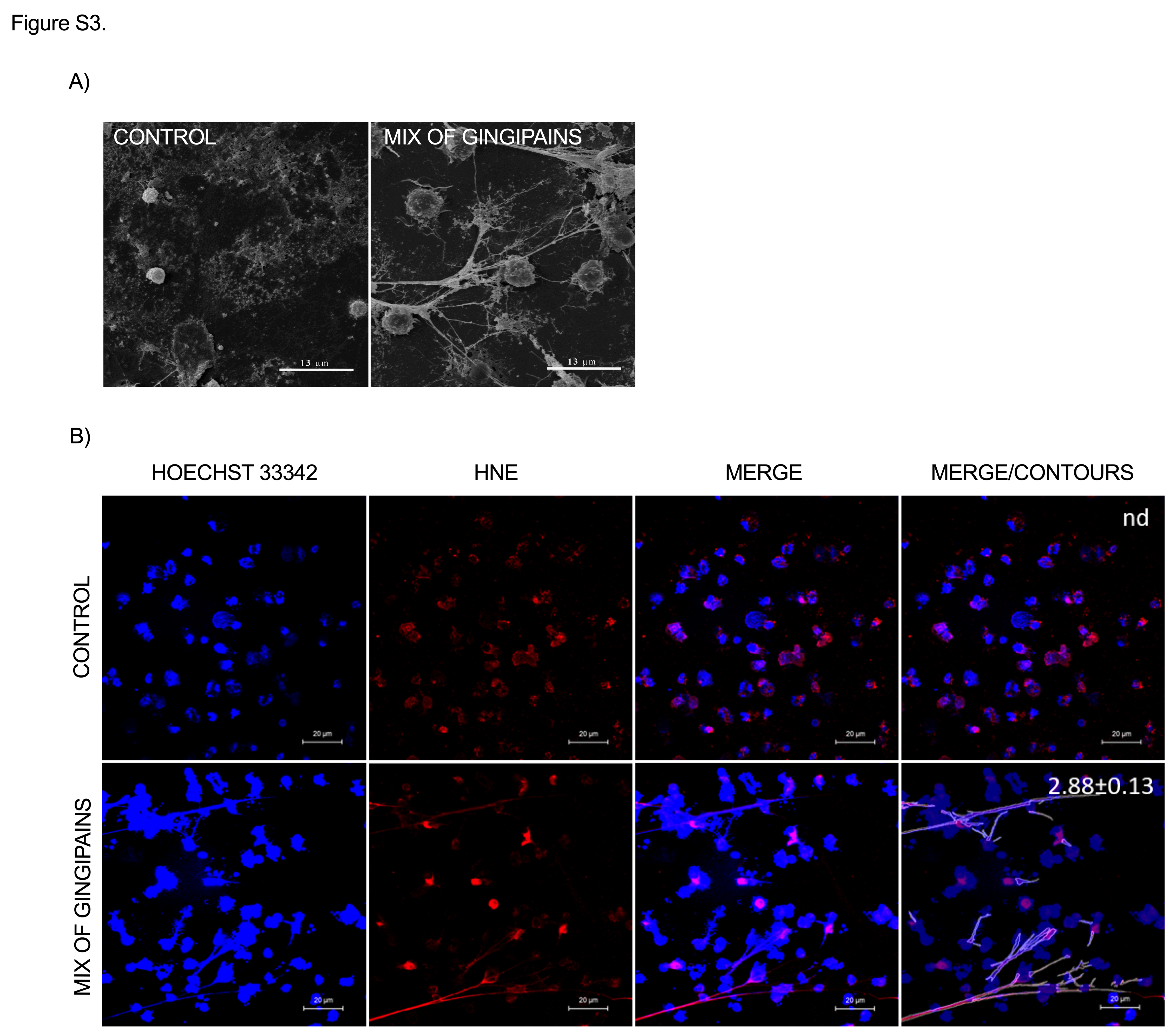

Supplement: S3 Fig — (A) Neutrophils were stimulated with an equimolar mixture of all three gingipains (RgpA, RgpB, and Kgp, each at 10 nM) for 4 h. NET structures were visualized by SEM. (B) For confocal laser scanning microscopy, DNA was stained with Hoechst 33342 (blue), and human neutrophil elastase (HNE) was stained with an APC-labeled antibody (red). Bars represent 20 μm. Quantitative analysis of NETs images was performed by merging blue and red channels (merge/contours). Percentage of the NET area in relation to the area of an image is presented as mean data (± SEM) from three independent images. n.d.- not detected NETs. (TIFF) [file ppat.1007773.s003.tiff]

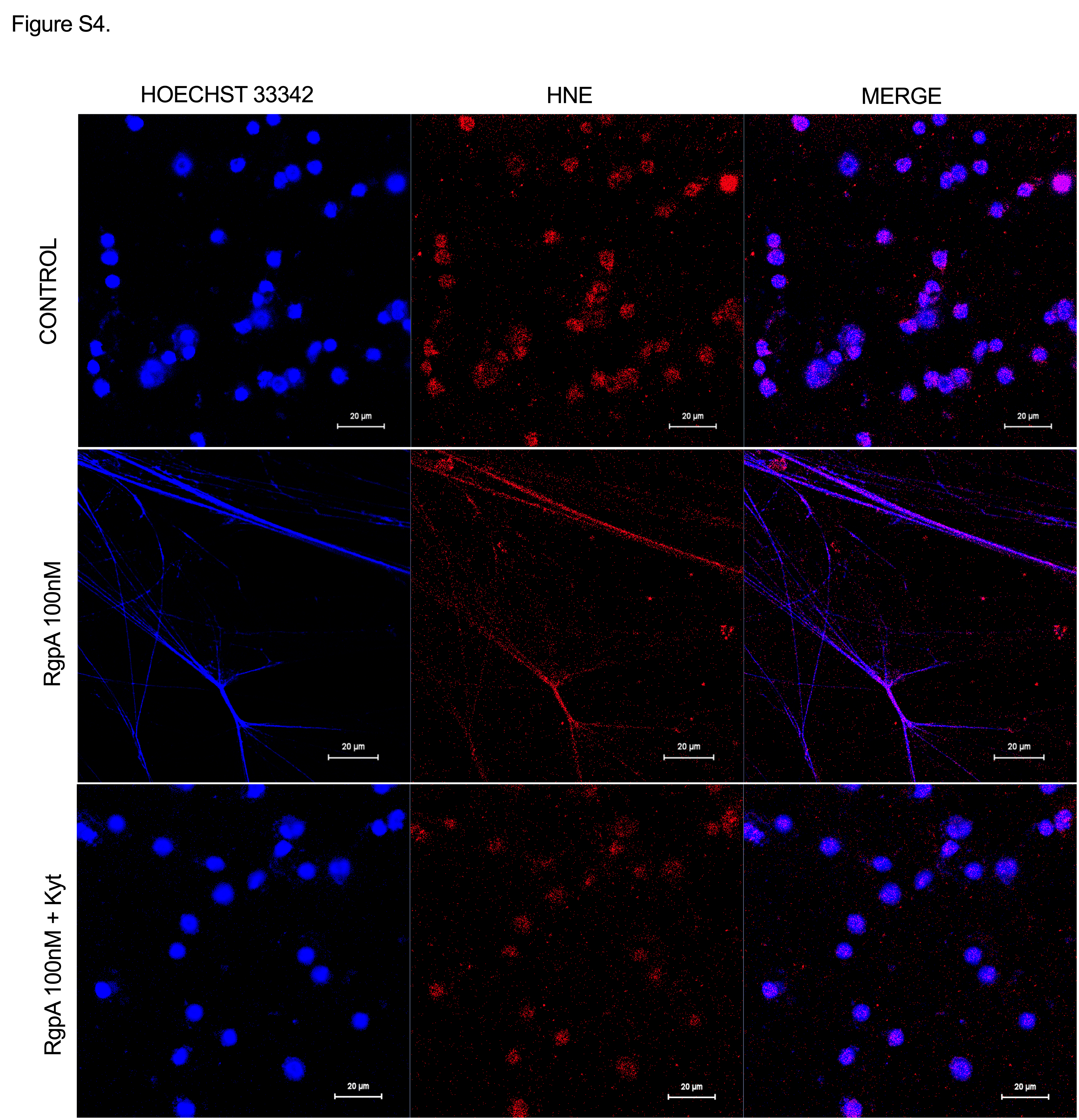

Supplement: S4 Fig — For confocal laser scanning microscopy neutrophils isolated from mouse peritoneal cavity were stimulated with 100 nM RgpA in the presence or absence of Kyt-1 at a final concentration of 1 μM. DNA is shown in blue (Hoechst 33342) and human neutrophil elastase (HNE) expression is shown in red. Bars represent 20 μm. (TIFF) [file ppat.1007773.s004.tiff]

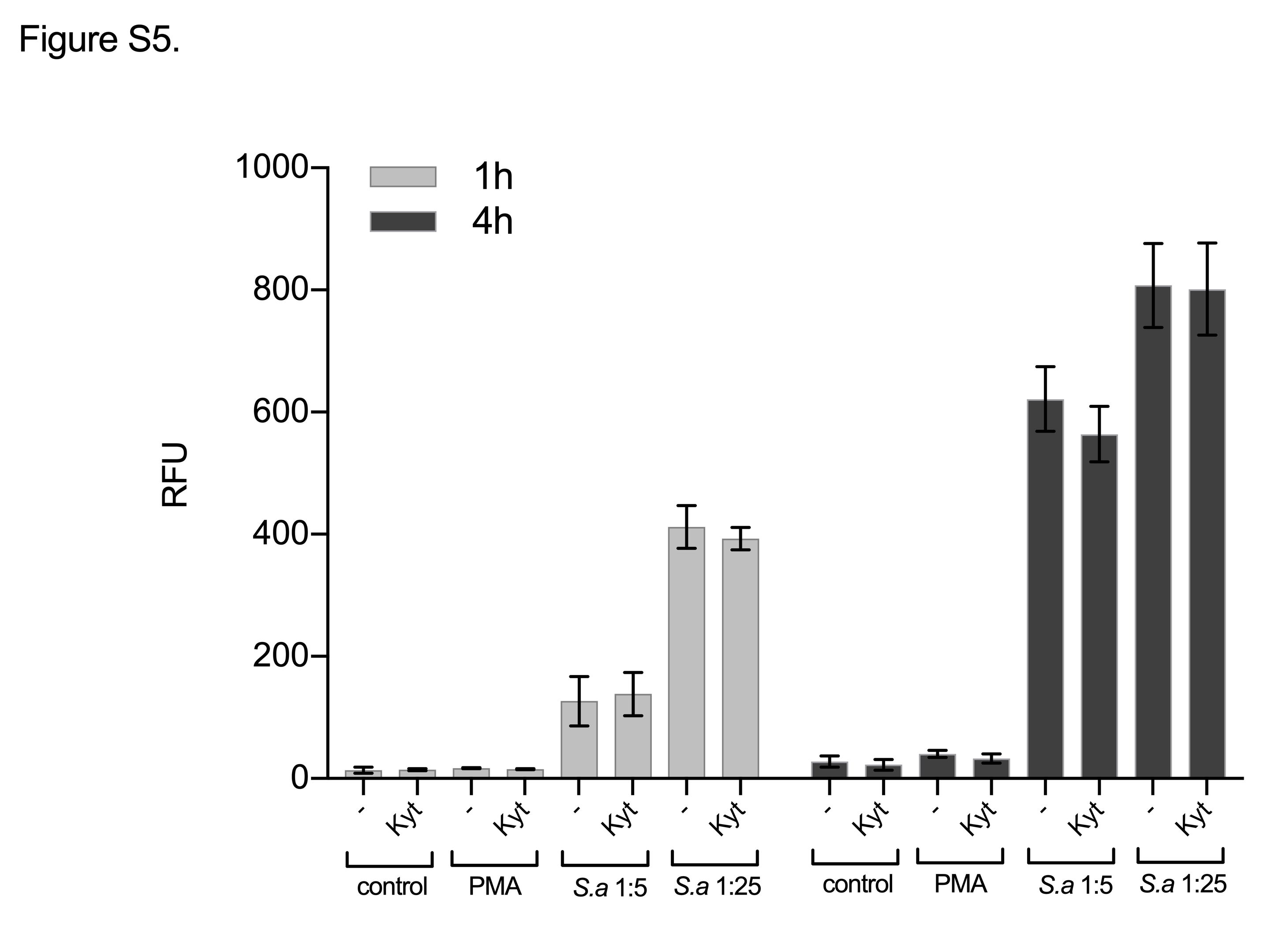

Supplement: S5 Fig — Human peripheral blood neutrophils were stimulated for 1h and 4 h with 25 nM PMA and S. aureus at MOIs of 1:5, 1:25 with or without pretreatment with Kyt-1 (1 μM). The level of NETs was determined by QPG. Mean data (± SEM) from a single experiment are shown. (TIFF) [file ppat.1007773.s005.tiff]

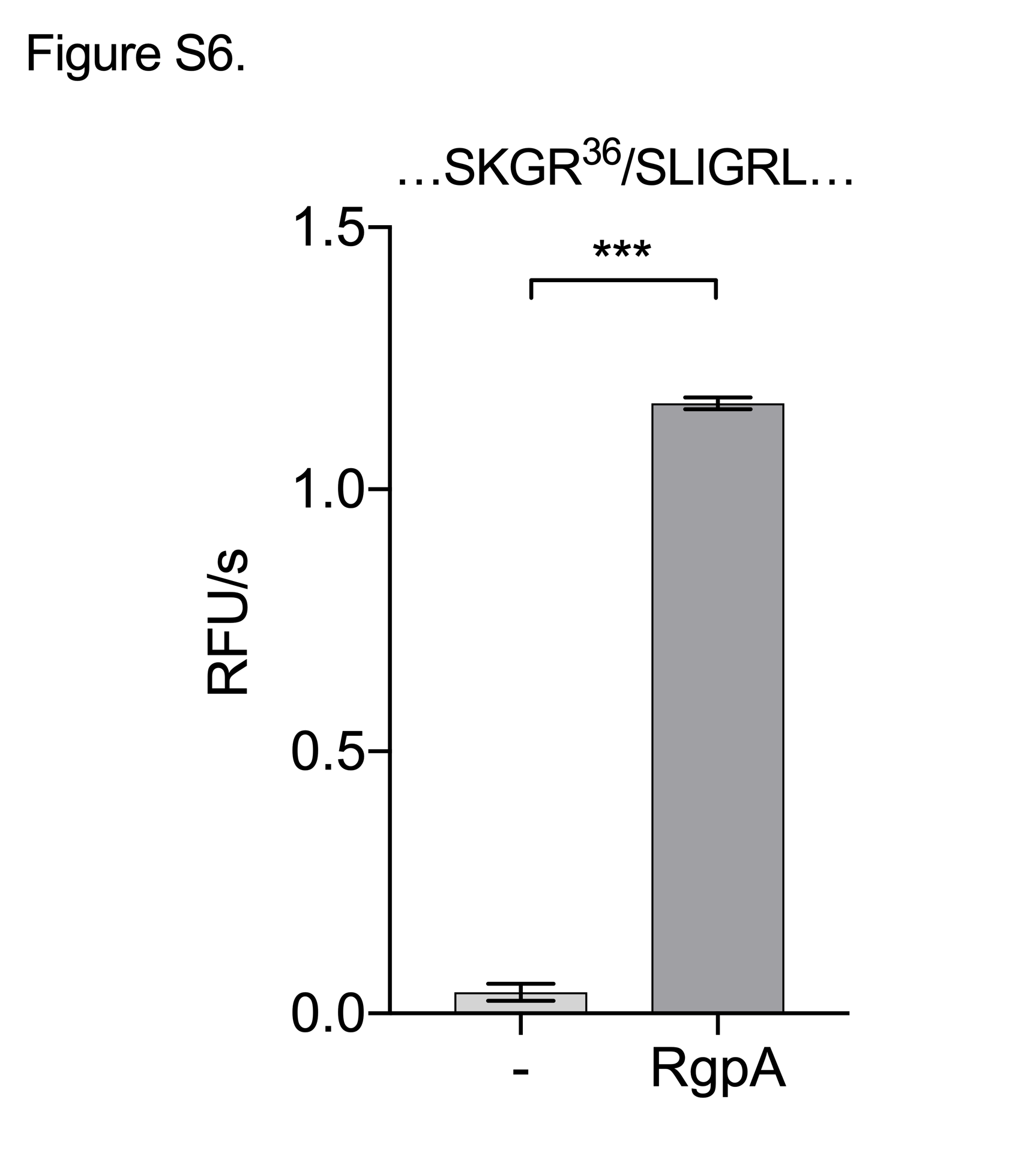

Supplement: S6 Fig — PAR fluorescence-quenched peptide (10 mM) were activated by 1 nM RgpA. The cleavage of PAR-specific sequences was estimated by fluorimetry and compared to the fluorescence background measured for the probe without RgpA. The canonical cleavage site is presented on the figure. Statistical significance was evaluated by unpaired t-test. Mean data (± SEM) from two independent experiments are shown. ***P < 0.001. (TIFF) [file ppat.1007773.s006.tiff]

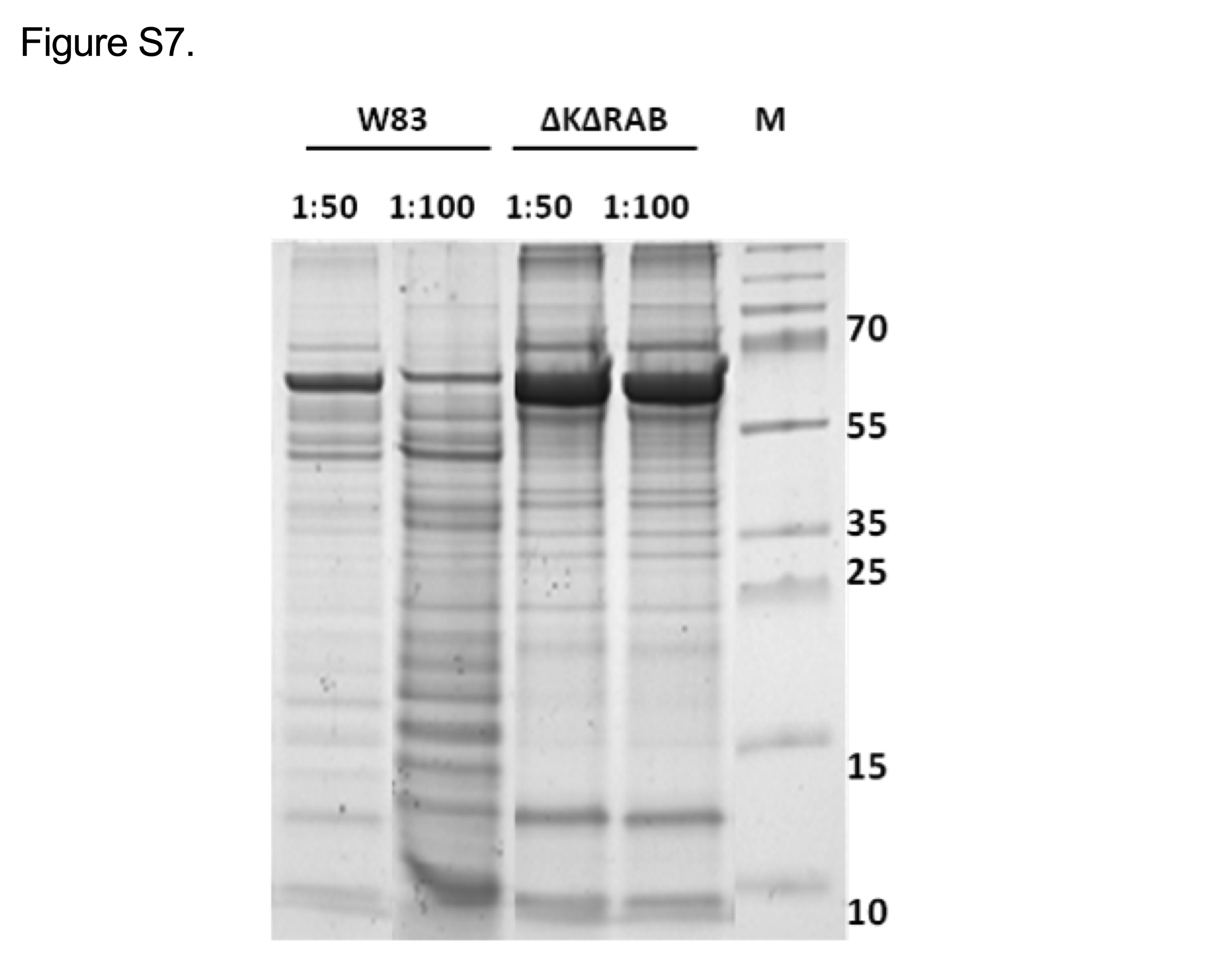

Supplement: S7 Fig — W83- and ΔKΔRAB-induced NETs (MOI 1:50 and 1:100) were collected 1 h after infection of neutrophils. Samples were separated by SDS-PAGE. A representative gel from one experiment is shown. (TIFF) [file ppat.1007773.s007.tiff]

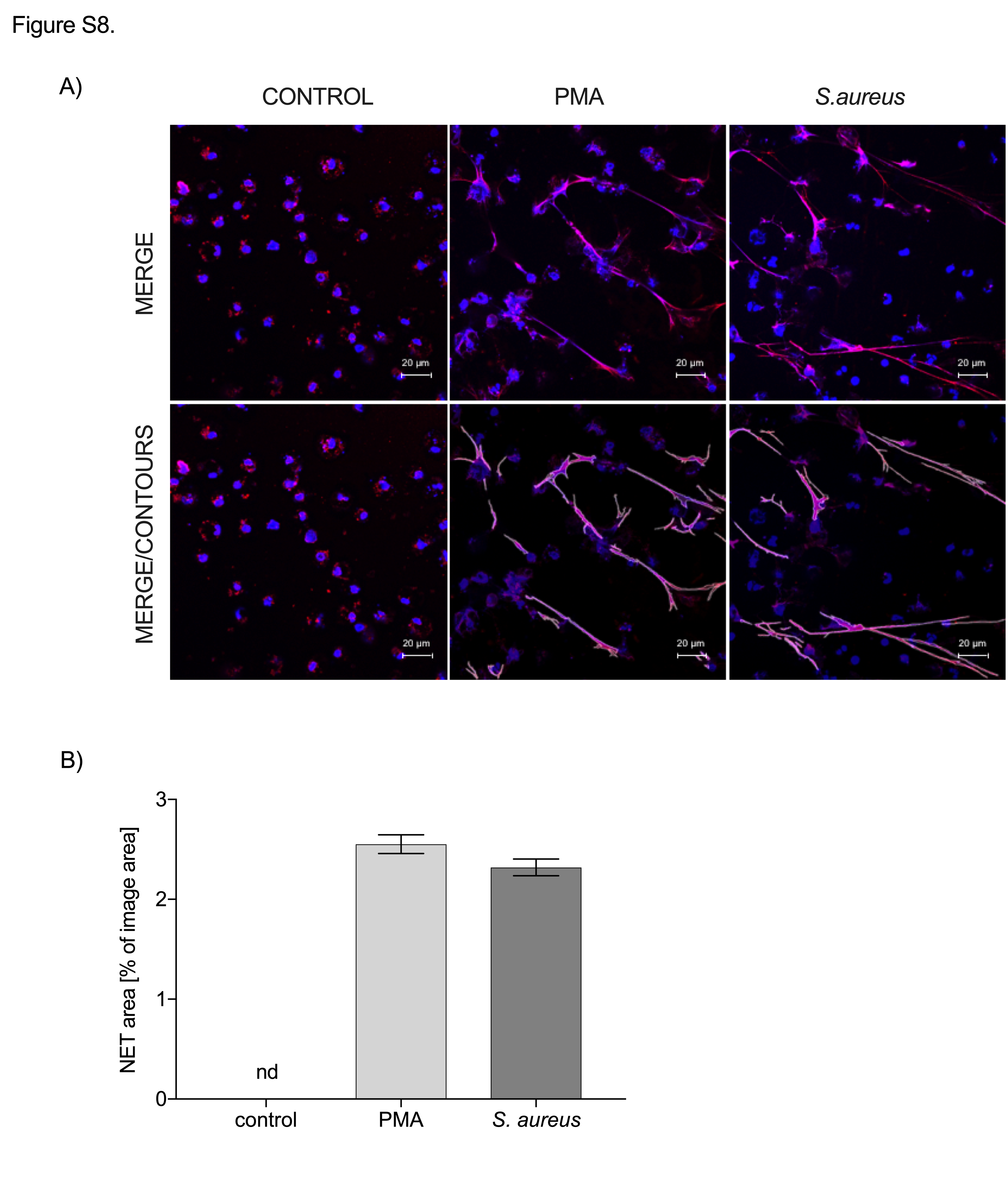

Supplement: S8 Fig — (A) For confocal laser scanning microscopy, DNA was stained with Hoechst 33342 (blue), and human neutrophil elastase (HNE) was stained with an APC-labeled antibody (red). Bars represent 20 μm. A representative quantitative analysis of NETs images by merging blue and red channels (merge/contours). (B) Percentage of the NET area in relation to the area of an image. Mean data (± SEM) from three independent images. n.d.–NETs not detected. (TIFF) [file ppat.1007773.s008.tiff]
